# ER stress induced immunopathology involving complement in CADASIL: implications for therapeutics

**DOI:** 10.1186/s40478-023-01558-1

**Published:** 2023-05-08

**Authors:** Mahmod Panahi, Yoshiki Hase, Xavier Gallart-Palau, Sumonto Mitra, Atsushi Watanabe, Roger C Low, Yumi Yamamoto, Diego Sepulveda-Falla, Atticus H Hainsworth, Masafumi Ihara, Siu Kwan Sze, Matti Viitanen, Homira Behbahani, Raj N Kalaria

**Affiliations:** 1 Department of Neurobiology, Care Sciences and Society, Center for Alzheimer Research, Clinical Geriatrics, Karolinska Institutet, BioClinicum J9:20 Visionsgatan 4, Solna, 171 64 Sweden; 2 Translational and Clinical Research Institute, Campus for Ageing and Vitality, Newcastle University, Newcastle upon Tyne, NE4 5PL UK; 3 Biomedical Research Institute of Lleida (IRBLLEIDA) - +Pec Proteomics Research Group (+PPRG) - Neuroscience Area, University Hospital Arnau de Vilanova (HUAV) - Department of Psychology, University of Lleida (UdL), Lleida, Spain; 4 Equipment Management Division, Center for Core Facility Administration, Research Institute, National Center for Geriatrics and Gerontology (NCGG), 7-430, Morioka-cho, Obu-shi, 474-8511 Aichi Japan; 5 Department of Molecular Innovation in Lipidemiology and Department of Neurology, National Cerebral and Cardiovascular Center, 6-1 Kishibeshinmachi, Suita, 564-8565 Osaka Japan; 6 Molecular Neuropathology of Alzheimer’s Disease, Institute of Neuropathology, University Medical Center Hamburg-Eppendorf, 20246 Hamburg, Germany; 7 Molecular and Clinical Sciences, St George’s University of London, Cranmer Terrace, London, SW17 0RE UK; 8 Faculty of Applied Health Sciences, Brock University, St. Catharines, ON L2S 3A1 Canada; 9 Department of Geriatrics, University of Turku, Turku City Hospital, Kunnallissairaalantie 20, Turku, 20700 Finland

**Keywords:** CADASIL, Complement, Interleukin 6, Intracellular adhesion molecule, Stroke, Vascular smooth muscle cells

## Abstract

Cerebral autosomal-dominant arteriopathy with subcortical infarcts and leukoencephalopathy (CADASIL) is caused by *NOTCH3* mutations. Typical CADASIL is characterised by subcortical ischemic strokes due to severe arteriopathy and fibrotic thickening of small arteries. Arteriolar vascular smooth muscle cells (VSMCs) are the key target in CADASIL, but the potential mechanisms involved in their degeneration are still unclear. Focusing on cerebral microvessels in the frontal and anterior temporal lobes and the basal ganglia, we used advanced proteomic and immunohistochemical methods to explore the extent of inflammatory and immune responses in CADASIL subjects compared to similar age normal and other disease controls. There was variable loss of VSMC in medial layers of arteries in white matter as well as the cortex, that could not be distinguished whether *NOTCH3* mutations were in the epidermal growth factor (EGFr) domains 1–6 or EGFr7-34. Proteomics of isolated cerebral microvessels showed alterations in several proteins, many associated with endoplasmic reticulum (ER) stress including heat shock proteins. Cerebral vessels with sparsely populated VSMCs also attracted robust accrual of perivascular microglia/macrophages in order CD45^+^ > CD163^+^ > CD68^+^cells, with > 60% of vessel walls exhibiting intercellular adhesion molecule-1 (ICAM-1) immunoreactivity. Functional VSMC cultures bearing the *NOTCH3 Arg133Cys* mutation showed increased gene expression of the pro-inflammatory cytokine interleukin 6 and ICAM-1 by 16- and 50-fold, respectively. We further found evidence for activation of the alternative pathway of complement. Immunolocalisation of complement Factor B, C3d and C5-9 terminal complex but not C1q was apparent in ~ 70% of cerebral vessels. Increased complement expression was corroborated in > 70% of cultured VSMCs bearing the *Arg133Cys* mutation independent of N3ECD immunoreactivity. Our observations suggest that ER stress and other cellular features associated with arteriolar VSMC damage instigate robust localized inflammatory and immune responses in CADASIL. Our study has important implications for immunomodulation approaches to counter the characteristic arteriopathy of CADASIL.

**Supplementary Information:**

The online version contains supplementary material available at 10.1186/s40478-023-01558-1.

## Introduction

Cerebral autosomal-dominant arteriopathy with subcortical infarcts and leukoencephalopathy (CADASIL) is recognized as the most common familial small vessel disease (SVD). CADASIL is caused by mutations of the *NOTCH3* gene on chromosome 19 [24]. The neuropathology of CADASIL is characterised by arteriopathy in perforating arteries, subcortical infarcts and deposition of granular osmiophilic material (GOM) in proximity to vascular smooth muscle cells (VSMCs), which contains fragments of the extracellular domain of NOTCH3 (N3ECD). The globular appearance and degeneration of VSMCs in CADASIL [58] in a segmental manner along perforating arteries [38] is the most distinct feature of CADASIL. Affected myocytes acquire variable nuclear sizes and shapes with abnormal chromatin appearance [12] that is related to the stress caused in the endoplasmic reticulum (ER) by accumulation of mutant NOTCH3 extracellular domain [36, 56]. Consistent with the degeneration of VSMCs, decreased expression of integrin beta1 subunit and beta1 immunoreactivity were reported in small arteries, arterioles and capillaries in CADASIL [11].

This was coupled with the decreased co-localization of NOTCH3 with autophagy markers LC3, and Lamp2 and increased levels of p62/SQSTM1 in VSMC (*Arg133Cys*) compared to the VSMC (wildtype)[22].

Changes within arterial VSMCs may also influence or activate the endothelium [21, 44] or modulate pericytes [50] although it is unclear how these are instigated. In addition, arterioles in CADASIL are associated with variable perivascular inflammatory infiltrates, not only in the brain but also in certain internal organs [45]. The pathogenesis of arterial pathology in CADASIL has been attributed to several pathways including VSMC apoptosis, [15, 63], autophagy [22] and low proliferation rates [23, 56, 62], which may be due to decreased trophic support e.g. transforming growth factor-β expression [41] and reduction in metabolism [42].

Suspected CADASIL cases have been linked to inflammatory or autoimmune mechanisms [43] prompted by clinical features including a relapsing-remitting course and widespread white matter lesions akin to those in multiple sclerosis, or vasculitis or inflammatory SVD [51]. It has previously been proposed that the presence of morphological changes similar to those described in panarteritis nodosa (PAN) VSMCs may be reminiscent of autoimmunological mechanisms [45]. However, various inflammatory and immune system markers have been described to be associated with features of CADASIL. In searching for an immune mechanism in the pathogenesis of CADASIL, Bousser and colleagues [3] had described in the first CADASIL case that whereas immunoglobulin (Ig) G, IgA, IgM, IgE, κ and α chains were not evident there was weak complement C3c, and Clq reactivity in arterioles. This was attributed to low antibody specificity associated with hyaline degeneration [17]. In fact, Gamble [17] had projected that deposition of iC3b within the walls of arterioles appears to be due to slow spontaneous activation of the alternative complement pathway and random binding of metastable C3b to proximate hyaluronic acid within the arteriolar wall. Similarly, in other studies [37], arteries exhibiting eosinophilic and periodic acid Schiff (PAS)-positive granules in the media showed complement-like but not IgG-, IgA, IgM, κ and ι chain-, or amyloid β-like immunoreactivities. In another CADASIL case diagnosed by the presence of GOM [47], cutaneous vessels were found with deposits of fibrin, complement and immunoglobulins. However, in a morphological study of a German family with CADASIL diagnosed by the presence of GOM in skin and brain vessels [6], typical PAS-positive granular degeneration did not react with antibodies against various immunoglobulins or complement factors. These findings collectively suggest dysregulated immune or complement responses associated with defective NOTCH3 signalling in CADASIL [5]. Furthermore, systemic oxidative stress and inflammation have been implicated in telomere shortening in peripheral blood leukocytes (PBLs) in CADASIL patients [46].

Given the foregoing, we still do not understand the extent of inflammatory or immune responses in CADASIL and whether degeneration of VSMCs together with endothelial and pericyte abnormalities is associated with bystander lysis possibly involving complement [48]. Furthermore, analogous to amyloid β plaques in Alzheimer’s disease (AD) that attract a repertoire of inflammatory and immune markers including complement [7], it is not certain whether GOM extracellular deposits are directly associated with inflammation. It is plausible that GOM could instigate a chronic inflammatory response or induction of complement within the arterial walls similar to that in atherosclerosis [28].

Based on the hypothesis that there is significant brain inflammatory response in CADASIL, we explored inflammatory and immune responses focused on cytokines and complement that might be associated with the severe arteriopathy. We performed various immunological investigations using post-mortem brain tissues and patient derived VSMC cell-lines of cerebral VSMCs in an attempt to elucidate key features of the immunopathology of CADASIL.

## Materials and methods

### Subjects and brain samples

Table 1 provides demographic details of all 55 subjects from which post-mortem tissues were obtained. The Table also shows diagnoses in 16 CADASIL and 11 similar age control subjects [8]. The mean age of CADASIL subjects was not different from the mean age of similar age controls (YC). Available case notes and radiological reports indicated that CADASIL subjects showed extensive WM changes consistent with small vessel disease (SVD) of the brain and met the minimum criteria for cognitive impairment [1, 55]. Majority of the CADASIL subjects had no known vascular disease risk [8]. The diagnosis of CADASIL was confirmed by the presence of *NOTCH3* gene mutations [64]. We also used groups of cases with SVD (n = 10) and similar age older controls (OC; n = 10) for comparison (Table 1). In addition, brain tissues from a Swedish family affected with PADMAL (n = 3) as well as from AD (n = 3) were used (footnote, Table 1). None of the controls had neurological or pathological evidence for cerebrovascular disease or neurodegenerative disorder.

**Table 1:** Demographic details and causes of death of CADASIL and other subjects

Parameter	Controls (Age-matched)	CADASIL	Old Controls	SVD‖
n (total = 55)	11	16	10	10
Mean age (years)	57.5	59.0	83.4	81.0
Range (years)	49–69	33–74	78–94	67–96
Gender (M/F)	6/5	10/6	2/8	5/5
Mean age at onset (years)	-	46.9	-	n/a
Duration of Disease (years)	-	12.6	-	n/a
**Key disease features**	.	Migraine, TIA, lacunar strokes, cognitive impairment	-	White matter disease, lacunar strokes, dementia
*NOTCH3* Mutations	-	*Arg133Cys* (6); *Arg141Cys* (2); *Arg153Cys* (2); *Arg169Cys* (3); *Cys445Arg* (1); *Arg558Cys* (1); *Arg985Cys* (1)	-	-
**Cause(s) of death**	Cardiac arrest, cancer, renal failure, infection	Bronchopneumonia, heart failure	Heart failure, cancer, ischemic bowel infection.	Heart failure, Cancer, GI bleed, sudden death
**Neuropathological Features**				
Braak stage	0	0	0-IV	1–4
CERAD score	1	0	2	0–2
Median Vascular Pathology score /10	3	9	5.5	6
Mean Sclerotic Index	0.28	0.36	0.27	0.30

Mean age of controls or young controls (YC) was not different to CADASIL and the mean age of the old controls (OC) was not different from SVD group. Mean age of OC group was different to YC and CADASIL (P < 0.05). No significant cerebrovascular or neurodegenerative disease pathology. Pathological findings not consistent with any neurological or psychiatric disease. The post-mortem interval between death and immersion fixation of tissue ranged 9–96 h. The length of fixation of tissues prior to paraffin embedding ranged 1 to 7 months. ‖In most of the subsequent quantitative analysis as indicated we used SVD cases (n = 8). Sclerotic index: *P < 0.05, different to YC; **P < 0.05, different to all groups. ANOVA and post-hoc analysis, †P < 0.41, CADASIL different from YC, old controls (OC) and PADMAL; ††P = 0.000, all groups; ‡P < 0.002, CADASIL was different to all groups, except PADMAL, but PADMAL was different to OC and SVD; ‡‡P = 0.000, CADASIL was different to all groups, except COL4A1, but COL4A1 was different to OC. Abbreviations: CADASIL, cerebral autosomal dominant arteriopathy with subcortical infarcts and leukoencephalopathy; CERAD, Consortium to Establish a Registry for Alzheimer’s Disease; GI, gastrointestinal; SVD, small vessel disease; PADMAL, pontine autosomal dominant microangiopathy with leukoencephalopathy (caused by collagen IV A1 UTR mutation in hereditary multi-infarct dementia of Swedish type); TIA, transient ischaemic attack

For immunohistochemical analyses, the brain tissues from CADASIL subjects (n = 16), SVD (n = 10) and controls (n = 20) were from the Newcastle Brain Tissue Resource (NBTR), Newcastle University, Campus for Ageing and Vitality, the Swedish Brain Bank (Stockholm, KI) and Brain Bank at Universidad de Antioquia, Medellin, Colombia. All studies involving human subjects have been approved by the local research ethics committee in Newcastle (REC 19/NE/0008); the NBTR committee, the Regional Ethical Review Board in Stockholm or the Research Ethics Committee of the South Huddinge University Hospital and Universidad de Antioquia, Medellin, Colombia.

### Immunohistochemistry, immunofluorescence staining and confocal microscopy

Table 2 provides details of the antibodies and semi-quantitative results obtained in this study. Immunohistochemical staining was performed on 10 or 20 μm thick paraffin sections. We assessed large sections containing the frontal and anterior temporal (pole) cortices and underlying white matter (Brodmann 9 and 21) and the caudate-putamen (basal ganglia) at the level of the anterior commissure [26]. Sections were dewaxed with xylene and re-hydrated in progressively declining concentration of ethanol. Antigen were unmasked by microwaving for 10 min in 10% citrate solution and quenched in 3% hydrogen peroxide. Non-specific antigens were blocked for 30 min at room temperature with either anti-horse serum or anti-goat serum depending on the origin species of where the primary antibody were generated from.

**Table 2:** Markers of Inflammation and Immunoreactivity in Cerebral Arteries and Arterioles in CADASIL

Marker (antibody, source)	Description and Source	Change in marker	CADASIL	Controls	Disease Controls† (SVD)
			(0, +(1),-++(2), +++(3) score of immunoreactivities or % (SD) of vessels positive for marker)		
**CADASIL Arteriopathy**					
**SMA**	α-smooth muscle actin (monoclonal, 1:3,000; Sigma)	↓Cytoskeletal component	+++	+	+++
**Fractin**	Actin filament fragment (polyclonal, 1:100, gift from Dr	↑ECM	+++	+	+++
**Medin**	Smooth muscle protein (polyclonal, 1:100, gift from Dr	↑ECM	+++	+	+++
**GLUT1**	Glucose Transporter 1 (polyclonal, 1:1,000; Thermo Scientific)	↓Endothelial cells	++	+	++
**COL1**	Collagen I (polyclonal,1:1,000; Dako)	↑ECM	+++	+	++
**COL3**	Collagen III (polyclonal, 1:1,000; Dako)	↑ECM	+++	+	++
**COL4**	Collagen IV (polyclonal, 1:1,000, Sigma)	↑ECM	+++	+	++
**Fibrinogen**	Fibrinogen (polyclonal, 1:2,000; Dako)	↑Plasma protein	++	+	+++
**Perivascular microglia/macrophages**					
**CD45**	CD45 (monoclonal, 1:1,000; Dako)	↑Microglial/macrophage cells	+++	+	+
**CD68**	CD68 (monoclonal, 1:400, Clone PG-M1, DAKO)	↑Microglial/macrophage cells	+++	++	++
**CD163**	CD163 (monoclonal, 1:1,000; Dako)	↑Macrophage cells	+++	-	+
**Iba-1**	Iba-1 (polyclonal, 1:1,000, 66827-1-Ig, Proteintech)	Microglial/macrophage cells	+++	+	++
**ER Stress proteins, UPS and cell receptors**					
**HSP70**	Heat shock protein 70 (polyclonal, 1:1,000; Dako)	Punctate deposits in arteriolar wall	+++	+	+
**HSP27**	Heat shock protein 27 (polyclonal, 1:1,000; Dako)	Degenerated VSMC in media	+++	+	+
**GRP78**	Glucose regulated protein 78 (polyclonal, 1:1,000; Dako)	Degenerated VSMC in media	+++	++	++
**ERp78**	ER stress-related proteins (CHOP and BIP/ERP78) (polyclonal, 1:100; Abcam)	Punctate deposits in arteriolar wall	+++	+	+++
**Calnexin**	ER stress marker (polyclonal, 1:100, AbCam)	Punctate deposits in arteriolar wall	+++	+	+++
**Ubiquitin**	Ubiquitin protease system (UPS) (polyclonal, 1:50; Dako)	Degenerated VSMC in media	++	+	++
**ICAM-1**	Intercellular Adhesion Molecule-1, CD54 (polyclonal, 1:300; Dako)	Reactivity in arteries and perivascular region	+++	+	++
**Cleaved Caspase 3**	Caspase-3 (polyclonal, 1:100, Cell Signalling Technology)	Punctate deposits in arteriolar wall	+++	++	+++
**Serum Amyloid Protein**	SAP (polyclonal, 1:1000; Dako)	Diffuse reactivity in arteries	+++	+	++
**Cystatin C (CST3)**	CST3 (polyclonal, 1:1000, Dako)	Diffuse reactivity in arteries	+++	+	++
NOTCH3 Signalling*					
**N3ECD**	A1-1 ab to NOTCH3 extracellular domain (polyclonal, 1:2,500; NILS [65]	↑N3ECD deposition	85.8 (4.9)	0	0
**N3ICD**	C2 ab to NOCTCH3 intracellular domain (polyclonal, 1:1,000 ; NILS [31]	↔N3ICD reactivity	85.4 (2.3)	82.3 (8.0)	80 (5.0)
**Complement System**					
**C1q**	Complement C1q (polyclonal, 1:1,000; Dako)		0	0	0
**C3d**	Complement 3 (polyclonal, 1:1,000; Dako)		75.6 (5.2)	0	0
**CFB**	Complement Factor B (polyclonal, 1:1,000; Serotec)		69.8 (6.3)	0	0
**C5-9**	Complement C5-9 complex (polyclonal, 1:1,000; Dako)		63.9 (2.7)	0	0

Table (last 3 columns) shows quantitative or semi-quantitative data derived from CADASIL cases, age-matched controls and cerebral SVD cases (as disease controls). Where percentages are not given, sections were scored from scale of 0 to 3, where + was designated as mild, ++ as moderate and +++ as frequent or abundant. Between 50–100 vessel profiles were viewed in each case involving arteries/arterioles in WM and overlying frontal cortex. Veins were avoided. †Vessels in three AD and PADMAL cases (age range 44–78 years) were also negative for complement. *Delta-1 and Jagged-1 were also examined. There was a tendency for increased activity in perivascular astrocytes (P > 0.05). Symbols: ↔, no change; ↑, increased; ↓, decreased or loss

Abbreviations: ab, antibody; ECM, extracellular matrix; AD, Alzheimer’s disease; PADMAL, pontine autosomal dominant microangiopathy and leukoencephalopathy; SD, standard deviation; SVD, small vessel disease

For immunofluorescence staining, formalin-fixed paraffin-embedded sections were autoclaved with antigen retrieval buffer (DV2004, DIVA Decloaker, Biocare Medical) for 30 min at 110 °C (Decloaking Chamber NxGen, Biocare Medical). After the temperature decreased to room temperature (RT), sections were washed with water for 5 min and then in Tris-Buffered Saline (TBS) + 0.05% Tween® 20 (TBS-T) (91,414, Sigma-Aldrich). The sections were blocked with Background Punisher (BP974, Biocare Medical) for 10 min at RT followed by washing and incubation with primary antibodies; α-smooth muscle actin antibody (abcam, αSMA, 1:500, Clone 1A4, abcam, U.S.A.), as an indicator for VSMC, A1-1 (N3ECD), and complement factors (Table 2), in TBS-T, overnight at 4 °C in a humid chamber.

After washing in TBS-T, sections were incubated (1 h at RT) with appropriate secondary antibodies [anti-mouse and anti-rabbit IgG (H + L)] conjugated to Alexa Fluor 546 or Alexa Fluor 488 (Invitrogen) at a concentration of 1:500 in TBS-T. To reduce auto-fluorescence, the slides were further washed in TBS-T (3 × 10 min) and incubated with or without Sudan Black B (199,664, Sigma-Aldrich), 5 min at RT. Sections were then washed in TBS-T and were mounted with DAPI Vectashield Hard Set (H-1200, Vector Laboratories) and the slides were stored at 4 °C. Sections from controls and CADASIL subjects were also incubated without primary antibody and used as negative control.

### Image capture, quantification and analysis

Tissue sections were examined using a laser scanning confocal microscope (LSM 510 META, ZEISS, or core Facility Bionut Microscopy), and images were acquired using the same settings (laser intensity, detector gain, and amplifier offset). Also, the tissues fluorescence staining was recorded sequentially in separate channels with Plan-Apochromate 20 × (NA, 0.8), 40 × (NA, 1.2) oil and 100 × (NA, 1.45) oil objectives. Image processing was performed with the included ZEN software. Cell quantifications were performed with Neurolucida. Fluorescence intensity was measured with the ImageJ 1.383 software (NIH, MA, U.S.A.).

Quantitative analysis of the total percentage of vessels immunoreactive to various antibodies assayed were performed by the manual counting of one hundred vessels from randomly chosen fields viewed under 2.5x magnification objective [9]. We focused on vessels of size 50–300 μm in diameter consisting largely of arterioles. The appropriate grey and while matter boundaries were delineated by haematoxylin or DAPI (immunofluorescence) background staining within tissue sections.

### Proteomic analyses of cerebral microvessels

Cerebral microvessels were isolated from frozen brains of CADASIL case and control (Table 1; Supplementary File 1, Figs. 1, 2, 3 and 4; cf. Table 3). After homogenization of 1 g of brain tissues, microvessels were purified by the glass bead column (Supplementary Fig. 1). They were immunostained with Von Willebrand Factor (VWF) and α-SMA to ascertain identity (Supplementary File 1, Figs. 1 and 2). Microvessels fractions were then solubilized in RIPA buffer (Supplementary Fig. 3). Each of the fractions was digested with the trypsin overnight and identified by nano-flow liquid chromatography (nanoLC) /mass spectrometry (MS). For identification of proteins in SEQUEST the UniProt human database downloaded on June 23, 2015, with 180,822 sequences and 71,773,890 residues was used. FDR was set at < 1%, trypsin was set as proteolytic enzyme, oxidation (M) and deamidation (NQ) were set as variable modifications and cabarmydomethyl (C ) was set as fixed modification. For protein identification precursor mass tolerance was set at 10 ppm and a reporter ion integrated tolerance at 20 ppm. Label-free quantification of proteins was based on spectral count as previously reported [16]. The most abundant proteins identified (spectral count > 20) were included in the performed analyses as indicated in Supplementary Table 1. Raw data from all the fractions are provided in Supplementary files 2–7. Further categorization of proteins and pathway analyses were achieved by the use of the specialized software FunRich, Panther and DAVID.

### Cerebral VSMC cultures

CADASIL patient-derived cerebral arterial VSMC (VSMC^R133C^; p.Arg133Cys) as well as control VSMC (VSMC^WT^) cell lines were established from post-mortem subarachnoidal branches of cerebral arteries (human cerebral arterial VSMC) by collagenase digestion [20, 23, 41, 57]. Initially, identity of VSMCs was ascertained by a number of methods including α-SMA expression [57]. Briefly, post-mortem brain samples were collected within 24 h of death and all cell cultures were planned and established immediately after obtaining the samples. Small blocks of brain tissue (1–2 cm × 1–2 cm) were cut, fragmented with razor blades and the tissues surrounding the vessels (diameter approximately 0.4-1 mm) were removed with sterile scalpels. The fragments were then transferred to tubes with ice-cold 20 mM HEPES in Dulbecco’s modified Eagle’s medium (DMEM) (Life Technologies, USA), centrifuged for 5 min at 500 × *g* at RT. After discarding the supernatant, tissues were resuspended in 0.05% collagenase/dispase mixture (100 µg/mL, Roche) in 20 mM HEPES in DMEM, and incubated for 5 min at RT. The tissues were homogenized usinga10 mL pipette for every 5 min and the undigested material was removed by centrifugation at 1000 × *g* for 10 min at RT. The pellets were washed with 10 mL of PBS (with calcium and magnesium) four times following resuspension in 10 mL complete culture medium containing 10% FBS 2 mM L-glutamine, 100 U/mL penicillin and 100 µg/mL streptomycin at RT. Approximately, 1.5 mL aliquots of the free vessels in solution were transferred into sterile 12-well cell culture plates and incubated at 37 °C and 5% CO_2_ in a humidified environment. The media were changed every 2nd day and once the vessels had settled down at the bottom of the wells (~ 3 days) observing out-crawling cells from the tissues and their confluency, the VSMCs were harvested using 0.25% trypsin for further culturing into 35-mm cell culture dishes (1:1.23 surface ratio) [19]. For partial immortalization, the cells were infected with a human papilloma virus construct E6/E7 at early passage (passage 1 to 3). The infection was verified by culturing the cells in the presence of G418 (Invitrogen, Auckland, NZ, USA) (400 *µ*g/mL) for a 10-day period. Primary cells (passage 1) were confirmed to be VSMC using the marker *α*SMA. After the viral infection (passage 2 to 5), all VSMC lines were screened negative for mycoplasma using the VenorGeM mycoplasma detection kit (Minerva Biolabs GmbH, Berlin, Germany) and with DAPI staining.

VSMCs were routinely cultured in DMEM]/F-12, GlutaMAX medium (Life Technologies, USA) supplemented with 10% heat inactivated fetal bovine serum (FBS), 1% Penicillin-Streptomycin and 1% L-glutamine. The cell lines were kept in an incubator with 5% CO_2_ at 37 °C. Passages of cells were matched for each experiment and were between 18 and 28. For the co-cultures with VSMCs, we used human aortic endothelial cells (ECs; Life Technologies) in M 200 medium with low serum growth supplement (ThermoFisher Scientific) [41].

### Real time quantitative PCR (qRT-PCR)

VSMCs were grown overnight in a 6-well plate chamber with confluence of 100,000 cells. The following day, cells were lysed with RIPA buffer (ThermoFisher Scientific, U.S.A.) and the quality of RNA was determined with RIN (RNA Integrity Number) of 10. cDNA was prepared using Taqman gene expression master mix (Applied Biosystem) and SuperScript VILO cDNA Synthesis kit (ThermoFisher Scientific, U.S.A.) according to the manufacturer’s protocol. Quantitative (q)RT-PCR was performed using costume format TaqMan fast plate (Applied Biosystems; No. 4,427,562, Rev C; https://www.thermofisher.com/order/taqman-files). The gene designations for control were: *HPRT1*-Hs99999909_m1 (endogenous control) and *GAPDH*-Hs99999905_m1 (endogenous control). All probes were used in duplicates with 30 ng of cDNA. Quantitative RT-PCR was performed on a 7500 Fast Real-Time PCR System (Life Technologies, U.S.A.). The expressions of genes were normalized to internal control HPRT gene and analysis was used comparing the normalized value of control cell line to the normalized values of CADASIL cell line. All the quantitative data were from three independent biological replicates for each experiment and the control value was normalized to 1.

### Immunoblotting of VSMCs

VSMCs (CADASIL and control) grown to 80% confluence were harvested by scraping from the plate after washing twice with PBS. Cells were collected by 5 min centrifugation at 300* g*, and lysed in lysis buffer (0.65% NP40, 10 mM Tris pH 8.0, 1 mM EDTA, 150 mM NaCl) containing protease inhibitor (100 ×, ProteaseArrest G-Biosciences). The total protein content was measured using the Pierce bicinchoninic acid (BCA) protein assay kit (Thermo Scientific). Laemlli sample buffer (2 × LDS, Sigma-Aldrich) was added to the samples and used for SDS-PAGE and immunoblotting as previously described [4].

### Quantification of complement in a co-culture system by qRT-PCR

See Supplementary file.

### Statistical analyses

Statistical comparison of values between groups was measured by one-way ANOVA followed by Bonferroni’s post-hoc test. Student *t*-test was used for two-group comparisons. *p*-values < 0.05 were considered significant. The results are representative of at least three independent biological replicates expressed as mean ± SEM. All univariate and multivariate analyses were performed using *IBM SPSS Statistics* software (version 23.0). Where applicable, a two-tailed *p*-value of < 0.05 was considered as the threshold for statistically significant differences or associations in all analyses.

## Results

### Features of arteriopathy in CADASIL

We examined tissue from 16 CADASIL patients and 11 similar age (YC) controls and 10 older controls (OC) (Table 1). As expected, CADASIL subjects exhibited the characteristic clinical and genetic features diagnostic for CADASIL compared to controls and SVD subjects. Neither the CADASIL nor SVD cases showed remarkable global atrophy compared to OC or YC group. SVD pathology incorporated all features including lacunar infarcts, microinfarcts, hemosiderin leakage, perivascular spaces, hyalinosis in arterioles and white matter attenuation in subcortical structures. Immunostaining with antibodies to pathological markers of AD confirmed the general absence of any significant amyloid β pathology or neurofibrillary tangle burden in CADASIL and the controls (Table 1). The overall ABC scoring for AD type of pathology was determined to be absent or low in CADASIL and SVD cases [34].

**Fig. 1:**
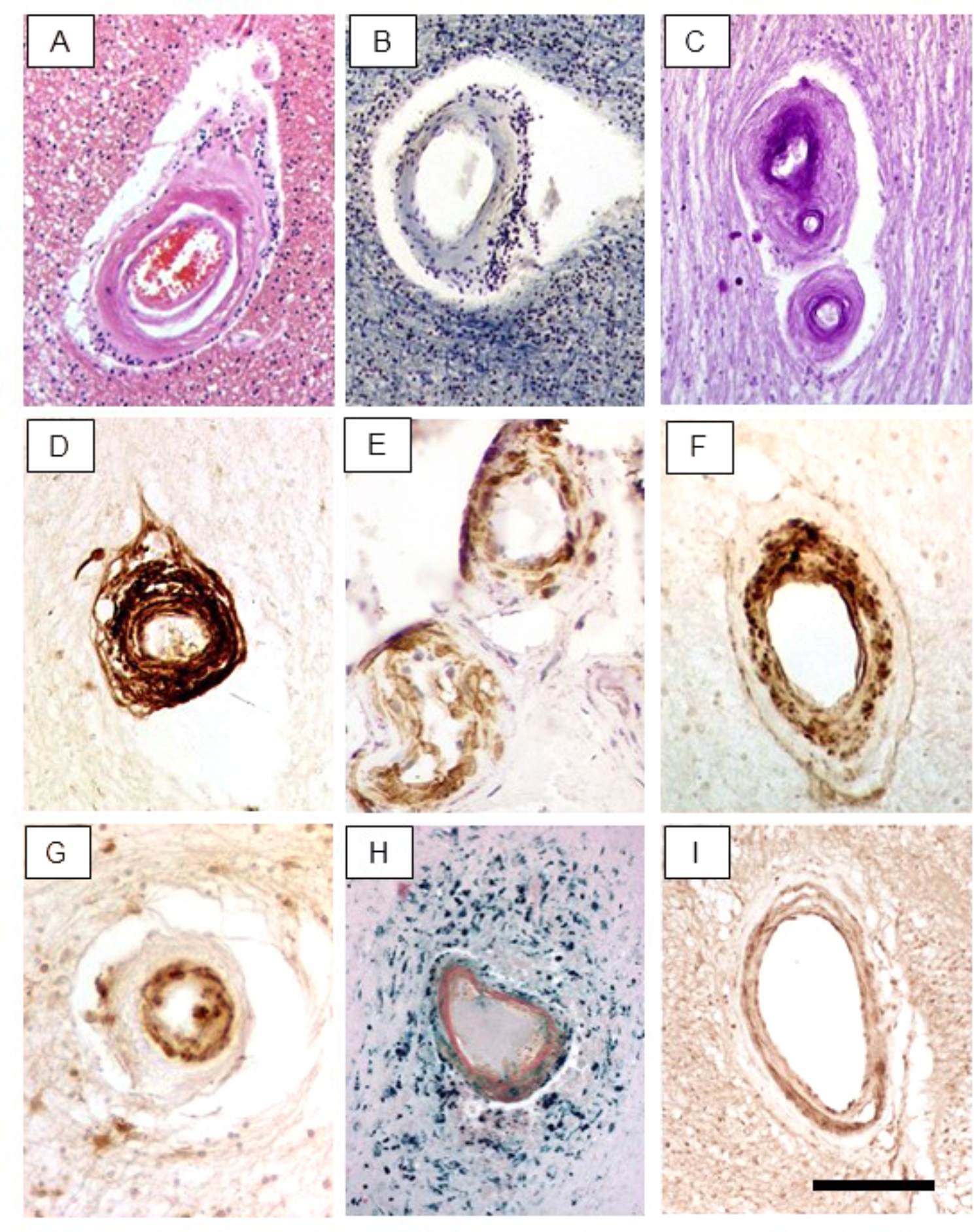
Cerebral arteriopathic changes in CADASIL. A-I, Representative images of arterioles in the deep WM showing differential degeneration of vessel walls. A-C, H, Sections stained with: H&E (A), LFB (B), PAS (C) and Perl’s (H) stain. D-G, I, Adjacent sections immunostained with antibodies to COL4 (D), αSMA (E), medin (F), N3ECD (G) and ubiquitin (I). Immunostaining with antibodies to fractin and amyloid P component was similar to medin (not shown). Note the differential changes in VSMCs and localization of N3ECD within intimal layers in the markedly hyalinised vessel (G). Perl’s stain (H) reveals perivascular iron (hemosiderin) deposits. Scale bar represents 200 μm (A-I)

In our sample (Table 1), we could not distinguish whether patients carrying *NOTCH3* mutations in the EGFr1-6 had greater burden of vascular pathology compared to those with mutations in the EGFr7-34 [27]. However, focusing on arteries and arterioles, we noted variable arteriopathy in CADASIL cases; medial-adventitial layers appeared to be affected foremost (Fig. 1). Subcortical structures exhibited greater arterial degeneration in all the cases compared to those in the cortex. Using various markers including αSMA, medin and fractin, we found VSMCs within the medial layers were variably disrupted, fragmented and atrophied (Fig. 1). These markers were present in walls of vessels with or without apparent perivascular spaces. In every CADASIL case, vessels were PAS^+^ and there was also evidence of calcification in 40% of the cases. Concentric lamination or onion-skinning, characteristic of severe hypertensive states often referred to as hyperplastic arteriolosclerosis was evident in several vessels within subcortical structures. Focal hemosiderin deposits were identified by Perl’s stain representing microbleeds in CADASIL. These were often seen around arteries indicating the presence of arteriolar leakage. In parallel with the profound disruption of arterial myocytes, evident in both the cytoplasm and nuclei, we also noted basement membrane thickening and increased immunoreactivity of collagen IV (COL4) in CADASIL although there may be different vascular modelling in SVD associated with hypertensive disease [30].

**Fig. 2:**
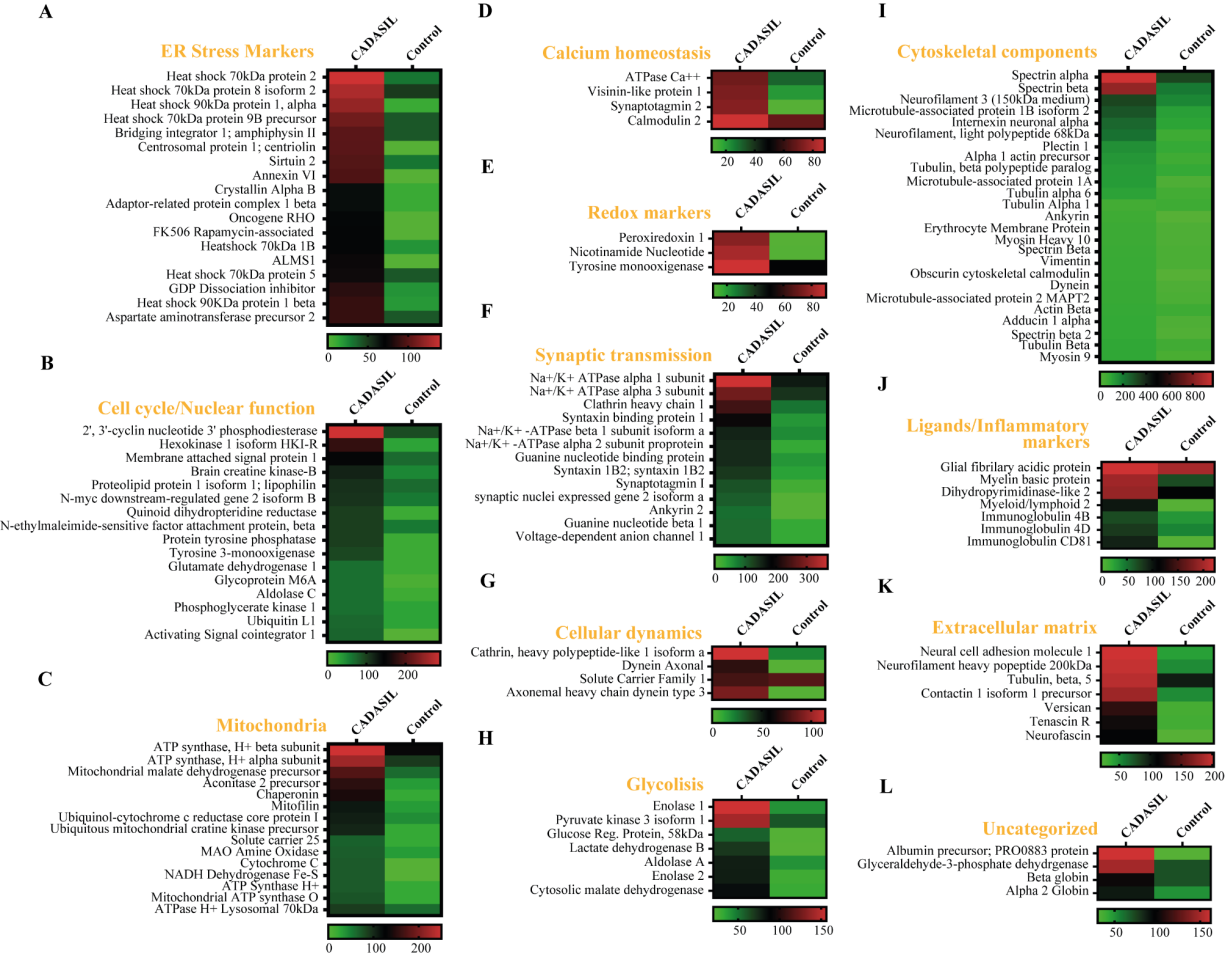
Heat map plots showing grouped categories of proteins with significant de-regulation differences in CADASIL versus YC. A, Proteins implicated in endoplasmic reticulum stress responses. B, Proteins implicated in cell cycle and/or nuclear responses. C, Proteins of the cerebral mitochondria proteome. D, Proteins implicated in cellular calcium homeostasis. E, Proteins implicated in antioxidant and oxidant responses. F, Proteins implicated in synaptic transmission, including ion channels and structural synaptic markers. G, Proteins implicated in cytoskeleteon-related intracellular dynamics. H, Proteins implicated in glycolysis. I, Proteins implicated in cellular structural functions and cytoskeleton. J, Proteins acting as integrins, ligands and inflammatory markers. K, Proteins of the extracellular matrix. L, Uncategorized proteins for the rest of the referred molecular and functional categorizations. All categorizations were based on specific proteomics categorization applications including FunRich, Panther and DAVID open software

We and others have previously reported on ER stress caused by mutant N3ECD [23, 36, 56] in CADASIL. Thus, we reasoned that this may lead to oxidative stress and inflammatory responses [18]. We attempted to demonstrate these changes by performing 2DGE proteomic analysis in cerebral microvessels, largely consisting of small arterioles and capillaries (Supplementary Fig. 1; Table 1). The RIPA buffer fractions revealed the highest number of detectable proteins, up to 875 and 896 in CADASIL and control samples but low numbers in the urea/thiourea and formic acid fractions. Protein abundance scores of > 20 (P < 0.01) ranged 26–279 and 29–301 in CADASIL and YC subjects in the three fractions, indicating high variability within extractable proteins in the microvessel samples (Supplementary File 1, Fig. 3). Consistent with the disruption of VSMCs, we found various myosin polypeptides, both muscle and non-muscle types and actin binding proteins e.g. adducin1 and obscurin. However, we found increases in proteins associated with the extracellular matrix such as COL4 and COL6 isoform. There was also evidence of severe disruption suggested by the abundant detection of an array of cytoplasmic, mitochondrial and nuclear proteins in CADASIL (Fig. 2).

**Fig. 3:**
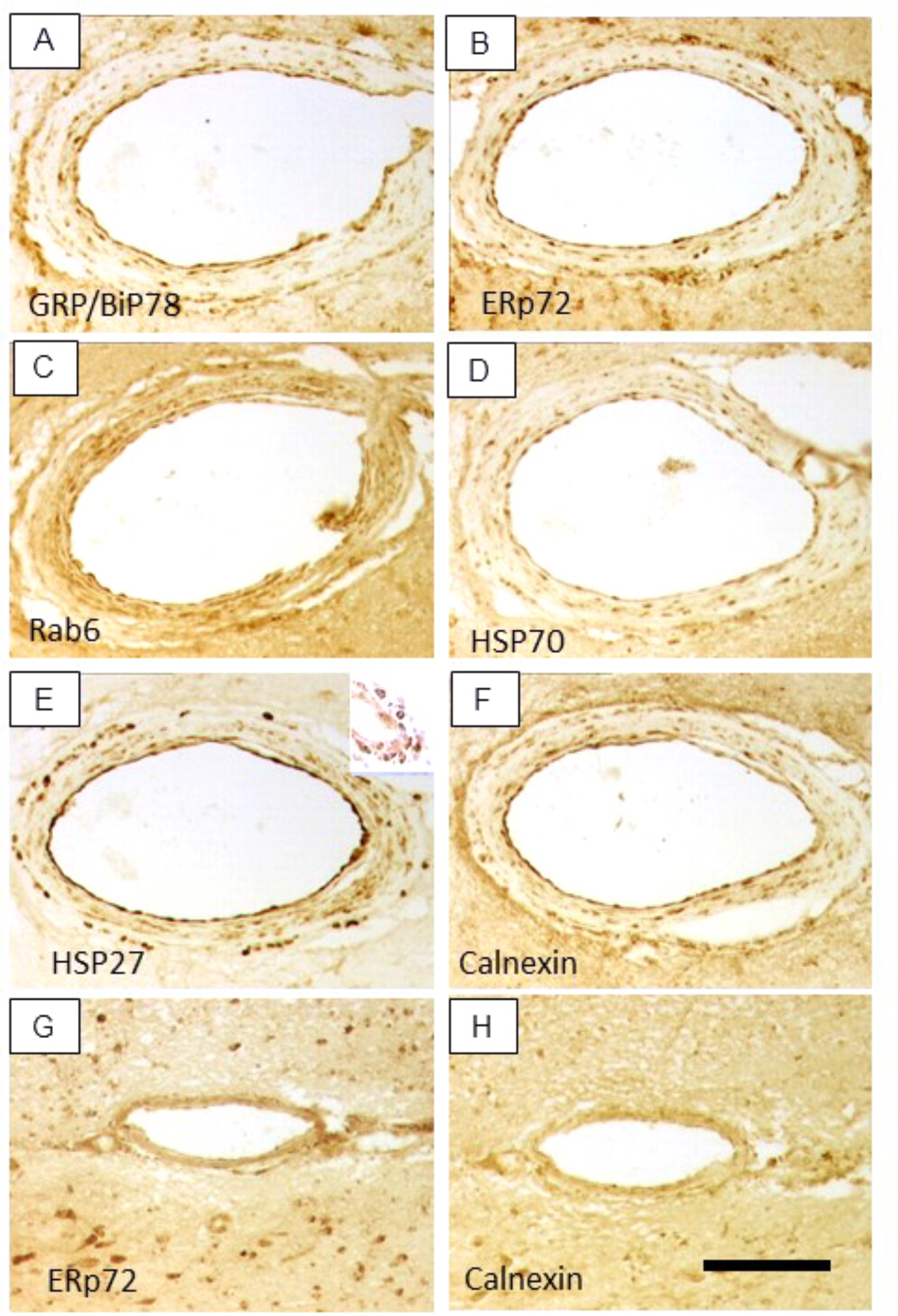
ER and UPR associated proteins in arterioles in CADASIL subjects. A-H, Representative images of the same blood vessel in the WM stained with antibodies to GRP/BiP78 (A), Erp72 (B), Rab6 (C), HSP70 (D), HSP27 (E), and Calnexin (F) in a CADASIL case and for Erp72 (G) and Calnexin (H) in similar age control. Mostly punctate immunoreactivity was evident in the arteriole wall in CADASIL, most marked for HSP27. Scale bar represents 150 μm (A-H)

Most importantly, the semi-quantitative analyses revealed increases in ER stress activated markers including galectin-1 and chaperones such as heat shock proteins (HSP) of which the 70 kDa type was most abundant in CADASIL (Table 2). In this context, we also noted that peroxiredoxin 1, an antioxidant enzyme and binding partner of guanine nucleotide binding protein (Gi2) and other unique markers of cellular stress such as FK506 binding protein 12-rapamycin associated protein 1 and ras homolog gene family were apparent in at least two CADASIL samples. Consistent with the calcium mobilization within arterial walls, calcium/calmodulin-dependent protein kinase and protein kinase C-γ were substantially decreased in CADASIL microvessels. We showed specific immunoreactivities of selected ER stress and nuclear ribonucleoproteins, where antibodies with high specificity were available (Fig. 3). We found immunoreactive deposits or punctate immunoreactivity within walls of moderately affected arterioles but not in YC subjects (Fig. 3; Table 2). Among these, HSP27 [54] exhibited the most robust immunoreactivity (Fig. 3E) apparent in ~ 50% of the vessels.

### Perivascular inflammatory cell responses

**Fig. 4:**
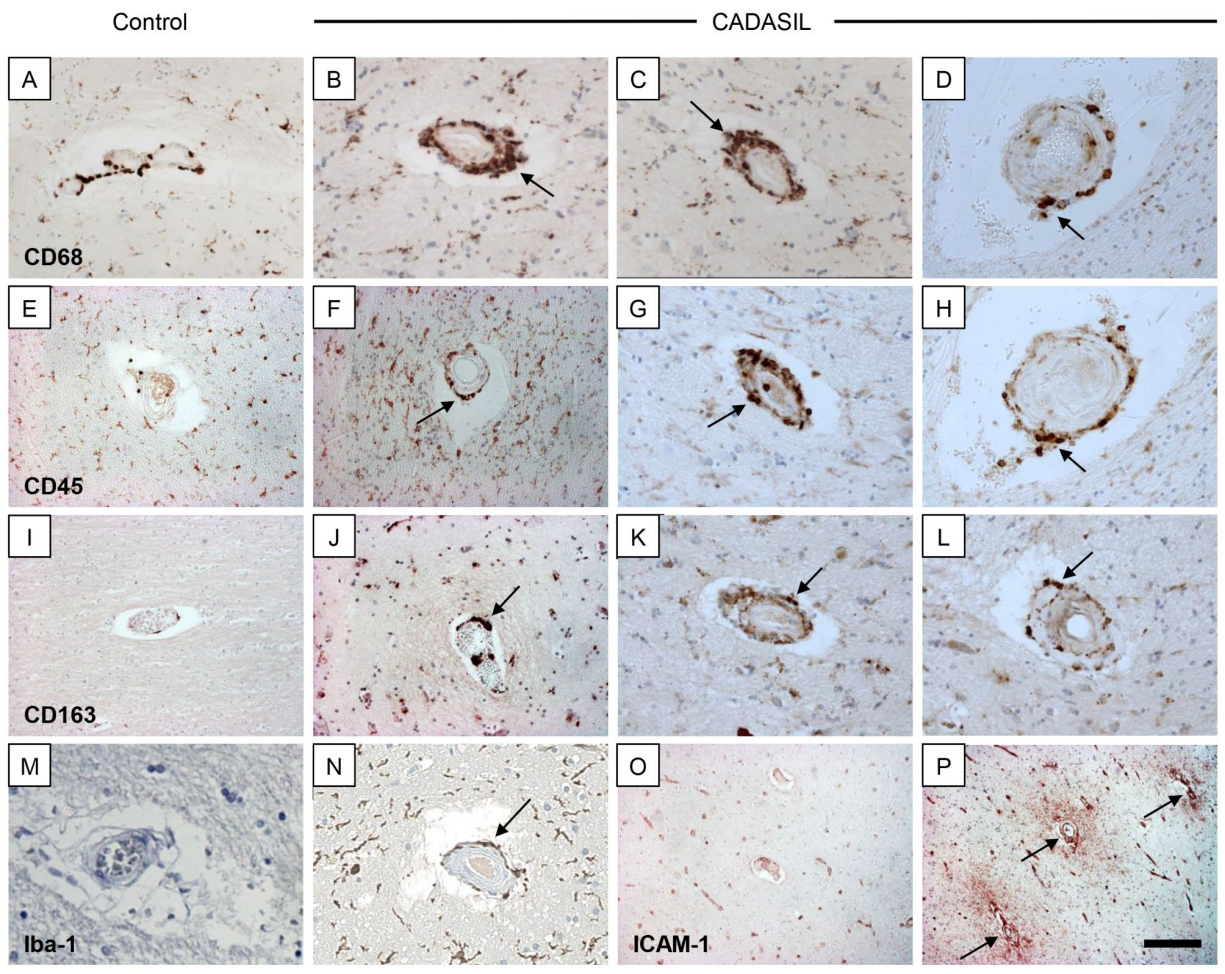
Perivascular cellular inflammatory responses in CADASIL. A-N, Differential CD68, CD45, CD163 and Iba-1 immunoreactivities in YC and CADASIL subjects O-P, ICAM-1 in YC and CADASIL subjects. Representative images of CD68 (A-D), CD45 (E-H), CD163 (I-L) and Iba-1 (M-N) in control and CADASIL subjects. A, E, I, M and O are YC; B-D, F-H, J-L and N show positive perivascular microglia/macrophages in serial sections from the deep frontal WM, basal ganglia and anterior temporal WM, respectively. The latter structures (C-D, G-H, K-L) showed intense perivascular inflammatory cells in CADASIL subjects. P, Intense immunoreactivity for ICAM-1 was evident in vessel walls in CADASIL but there was also diffuse reactivity in the perivascular parenchyma. Perivascular CD45 immunoreactivity and CD163, but not CD68, were increased in CADASIL subjects in all three regions compared to similar age controls *YC) (P < 0.05). Scale bar represents 200 μm (A-N) and 50 μm (O-P)

To explore the nature of inflammatory responses in the arterial walls, we first examined a repertoire of microglial and macrophage markers in arterioles in CADASIL subjects. In many vessels and particularly those with some remnant VSMC, we found constant presence of both activated CD68^+^ cells and CD45^+^ cells, with mostly perivascular distribution in all the three brain regions investigated (Fig. 4). The presence of CD45^+^ cells exceeded that of CD68^+^ by ~ 40% in CADASIL cases compared to YC subjects (P < 0.05). Mean (SEM) % area of immunoreactivities of CD45 was 0.6 ± 0.05 versus 0.84 ± 0.06 in YC and CADASIL subjects (Supplementary Fig. 5). These observations were also corroborated by 50% increased ratio of CD45^+^ to CD68^+^ cells in CADASIL compared to YC (P < 0.05). Both CD68^+^ and CD45^+^ cells were more prominent and concentrically distributed on vessel walls within perivascular spaces rather than the detached parenchyma. Iba-1^+^ cells showed similar distribution as CD68^+^ cells and they were similarly activated with greater numbers of Iba-1^+^ reactive cells in CADASIL compared to the controls. Remarkably, in CADASIL cases we also observed increased infiltration of CD163^+^ cells, which always had rounded amoeboid morphology, predominantly located in perivascular regions (Fig. 4). The unique presence of CD163^+^ cells as scavengers of haptoglobin-haemoglobin complexes [14] could relate to the presence of hemosiderin or haemoglobin from fragmented erythrocytes. We further showed that these inflammatory cells did not contain N3ECD immunoreactivity (Supplementary Figs. 6–7) but other macrophage-like cell types and pericytes tend to exhibit NOTCH3 fragments [65].

To assess, whether mediators of inflammatory cellular traffic across the cerebral endothelium are involved, we immunostained tissue sections for ICAM-1. We found profound ICAM^+^ immunoreactivity within vessel walls as well as within the perivascular regions (Fig. 4). There was consistently more reactivity in the media rather than the endothelium, which is also altered given thrombomodulin [21] was robustly increased in CADASIL. However, we did not specifically detect ICAM-1 in the proteomic profiles of cerebral microvessel (Supplementary Table 1).

### Pro-inflammatory markers in CADASIL VSMC

**Fig. 5:**
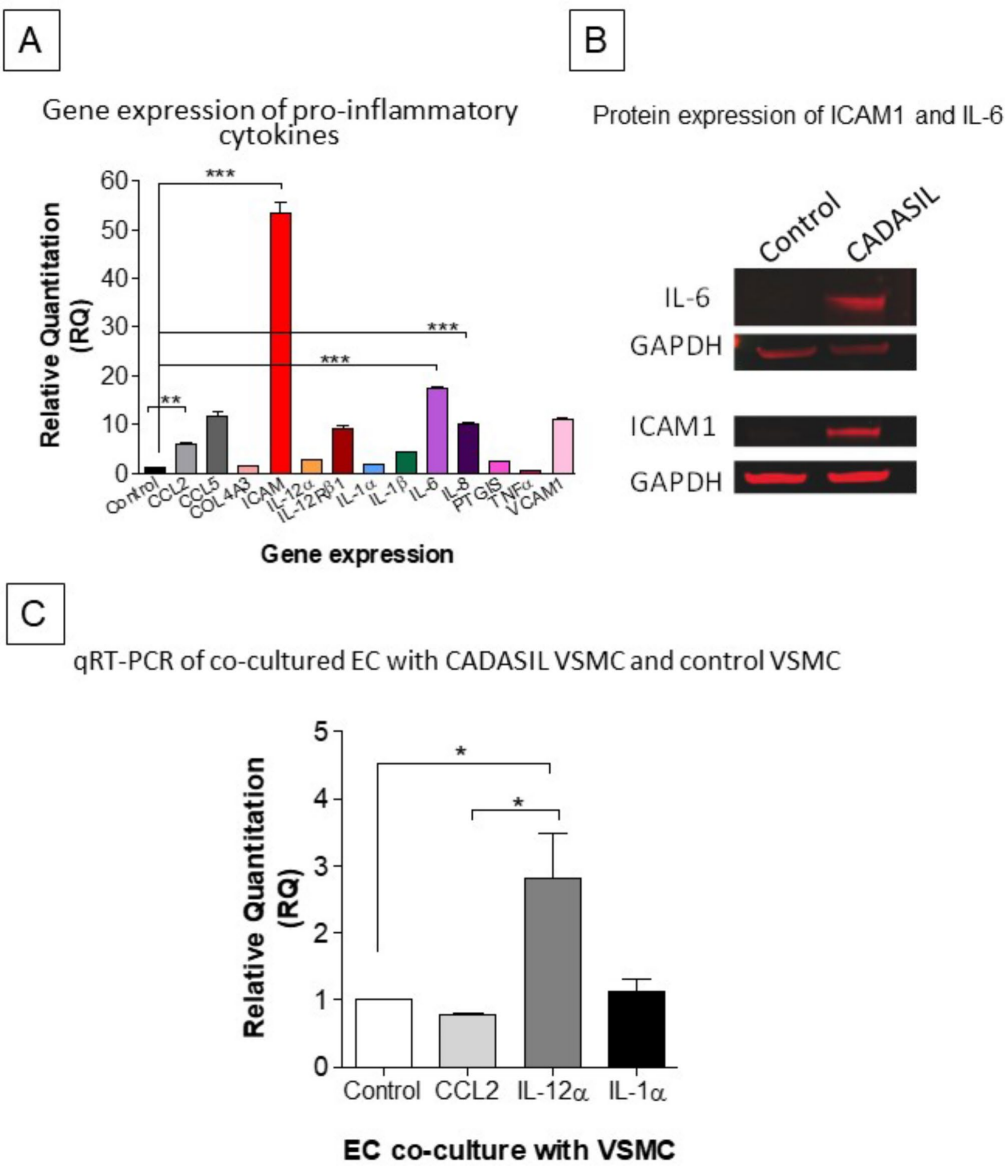
qRT-PCR and Immunoblotting analyses of ICAM-1 and inflammatory genes in CADASIL VSMCs. A, Plots show expression of several inflammatory genes in VSMCs in CADASIL Patient-derived cerebral arterial VSMC (VSMC^*Arg133Cys*,^*n = 1*) as well as control VSMC (VSMC^*WildeType*,^*n = 1*) cell line. Expression was normalized to the endogenous control gene; HPRT1, and the RQ (Relative Quantitation) was calculated using control VSMC normalized to 1. ** *P* < 0.05, **P < 0.01, ***P < 0.001. One-way ANOVA followed by Bonferroni’s post-hoc test was used for statistical analysis. B), Immunoblotting analyses were conducted to assess the protein expression of IL-6 and ICAM in VSMCs. The samples were visualized using the Odyssey CLx Imager. 15 µg of protein was loaded into the gel and GAPDH was used as loading control. C, Quantitative RT-PCR analysis of inflammatory gene expression in ECs co-cultured with CADASIL OR wildtype VSMC. Analysis showed increase in IL-12α and IL-1α gene expression in the ECs after they were co-cultured with Human Cerebral VSMC from CADASIL patient (*P < 0.05). The results are representative of three independent biological replicates (n = 3)

To test whether brain arterial myocytes in CADASIL patients produced inflammatory cytokines or integrins, we next performed qRT-PCR experiments in cerebral VSCMs cells harvested from patients with the *Arg133Cys* mutation, which was the most common genotype in our cohort. Since the list of pro-inflammatory cytokines is vast, we selected *ICAM-1* and several other genes that were previously identified in either stroke, cardiovascular disease, or other artery related diseases. We found that almost all the selected cytokines were upregulated in CADASIL VSMCs compared to control (Fig. 5A). The most upregulated genes were *ICAM-1* > *Il-6* > *Il-18, VCAM-1 > CCL5 > IL-12α*. Gene expression of the adhesion molecule *ICAM-1* and pro-inflammatory cytokine *IL-6* were increased by 50 and 16-fold, respectively (P < 0.01). To ascertain whether these findings were reflected in protein concentrations, we performed immunoblots. We focused on ICAM-1 and IL-6. Both IL-6 and ICAM1 showed high protein expression in CADASIL VSMCs compared to control VSMCs (Fig. 5B). These data suggested that the mutation that causes a frail VSMC in CADASIL also causes activation of inflammatory signals from these cells.

### Co-culture of CADASIL VSMC affects endothelial cells (ECs)

To study the functional consequences of elevated cytokines, we co-cultured control and CADASIL VSMC with endothelial cells (Fig. 5C). Since we intended to assess effects of secreted cytokines, we used trans-well plates so there would not be direct contact between VSMC and endothelial cells. Endothelial cells cultured in the presence of CADASIL VSMC exhibited high expression of IL-12α and IL-1α. This suggested CADASIL VSMC induce inflammatory responses in neighbouring endothelial cells thereby possibly influencing the circulating macrophages and lymphocytes into the parenchyma.

### Vascular immunopathology of components of the complement cascade

**Fig. 6:**
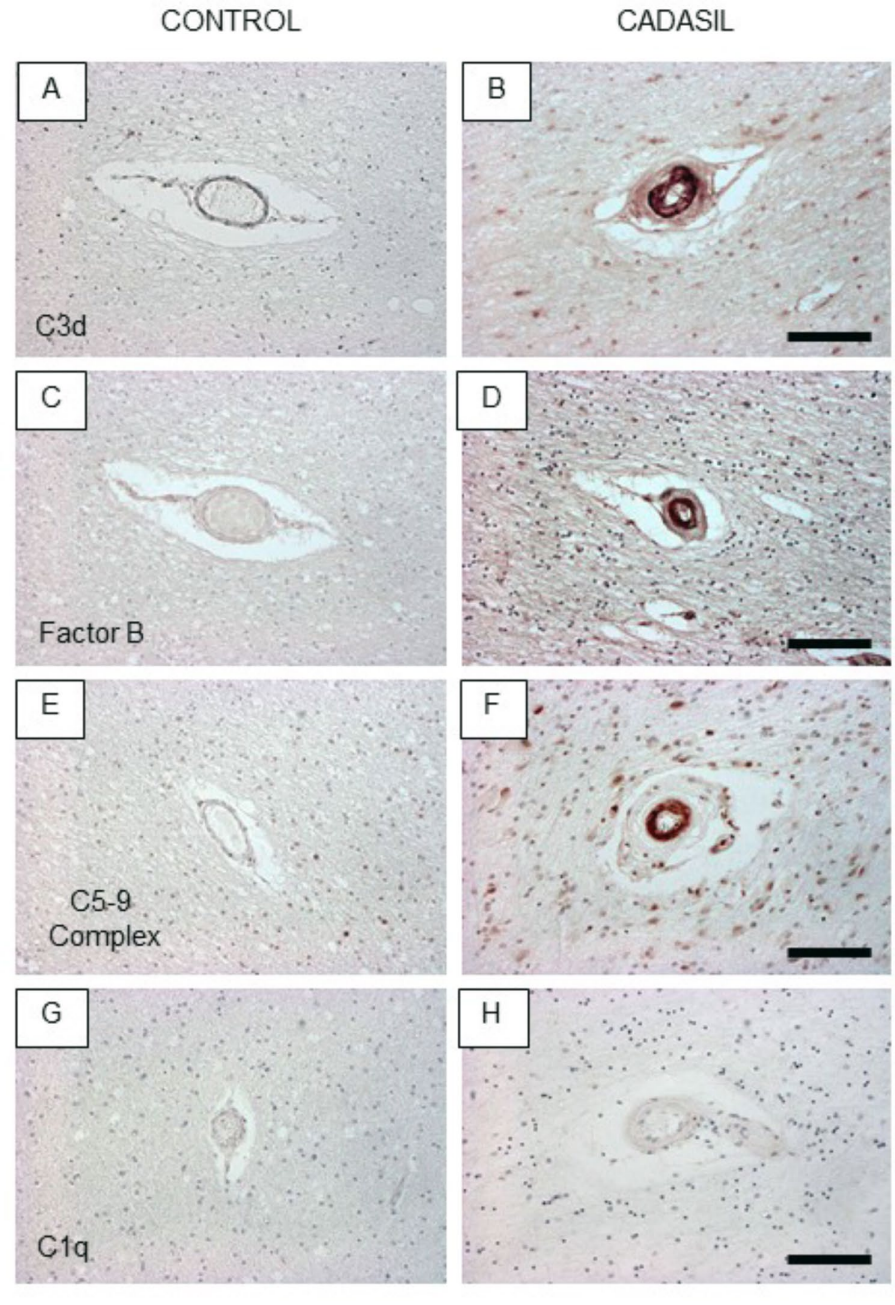
Activation of the alternative pathway in arterioles in CADASIL. A-F, Representative images of C3d (A, B), Factor B (C, D) and C5-9 complex (E, F) immunostaining in the temporal white matter of Control (A, C, E) and CADASIL (B, D, F). Lack of C1q immunoreactivity in CADASIL, C3d (B) and Factor B (D) were upregulated, and C5-9 complex (F) was observed in vessels compare to control. Scale bars represents 100 μm (B, D, F)

**Fig. 7:**
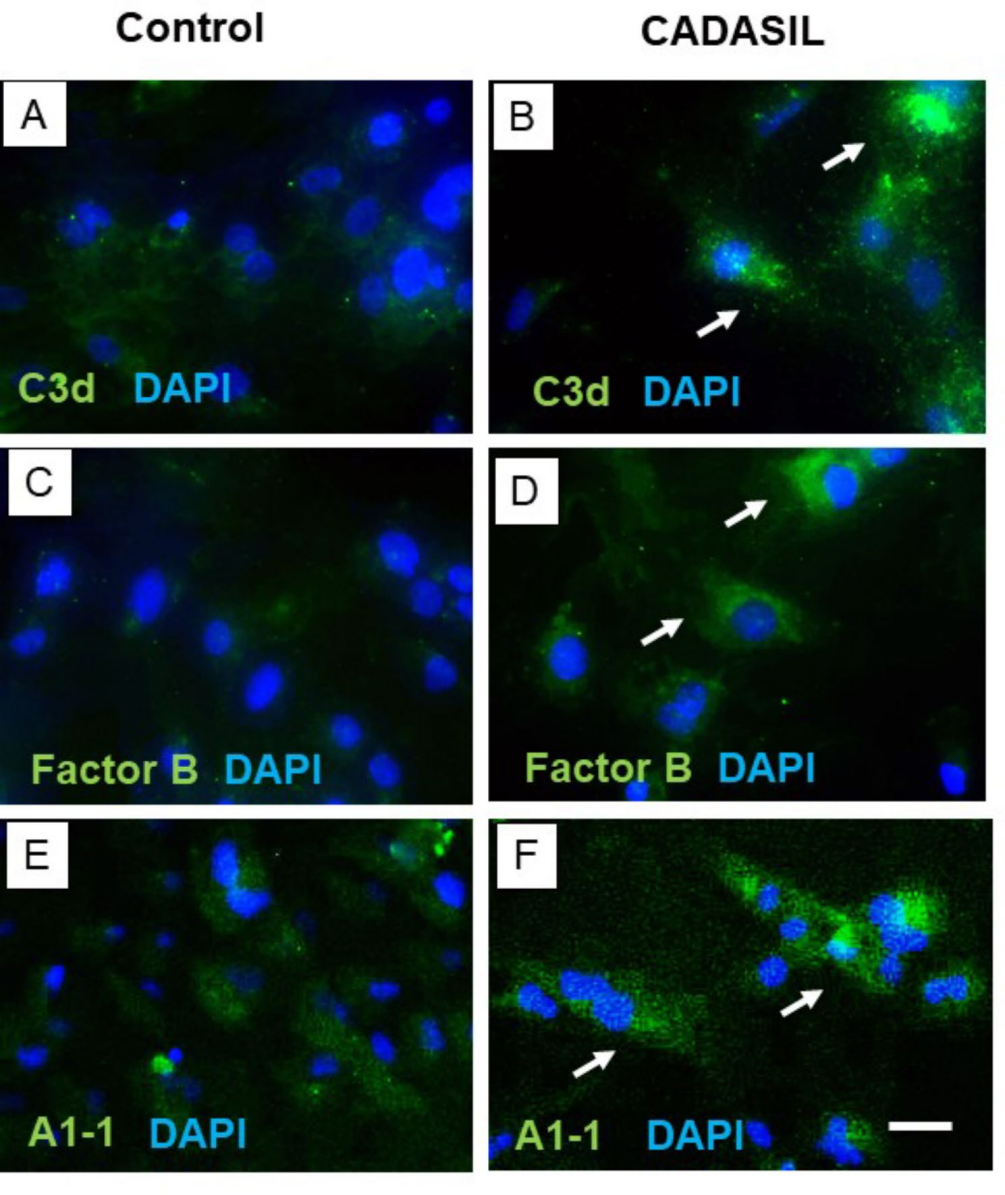
Complement C3d and Factor B in relation to N3ECD in VSMCs from CADASIL subjects. A-F, Representative immunofluorescent images showing specific C3d (A, B) and Factor B (C, D) localization in VSMCs from control and CADASIL subjects. Specificity was also demonstrated by the localization of N3ECD reactivity in VSMCs in CADASIL but not in control VSMCs. Arrows show positive reactivity in VSMCs in CADASIL. Quantification showed that C3d and Factor B immunoreactivites were localised in > 70% of the cells (n = 3 wells). There was no apparent co-localisation of complement and N3ECD, which was always distally located [57]. Scale bar represents 15 μm (A-F)

To ascertain if the observed inflammatory responses activated immune pathways, we next focused on the complement cascade. This would substantiate our previous observations in cerebrospinal fluid of CADASIL patients [59]. This could indicate that complement is activated by N3ECD fragments. Given there were indications of complement C3 in 2 previous proteomic studies in CADASIL (Table 3), we first looked for various complement pathway components to determine their activation. In addition to complement factor B, various other key components of the complement cascade including complement factors C1q, C3d and C5-9 (Membrane Attack Complex) were examined (Fig. 6; Supplementary Fig. 8; Table 2). Immunohistochemical staining revealed intense immunoreactivity of complement factors B, C3d and C5-9 in CADASIL patients compared to the age-matched YC groups. Of these 3 key components of the alternative complement cascade, C3d exhibited the strongest immunoreactivity (Fig. 6). There was also no appreciable immunoreactivity of these complement factors in any of the SVD control groups (Table 2). While there was variable background reactivity of these factors in other cells, including neurons, microglia, astrocytes and oligodendrocytes, there was no specific trend, and such staining was considered to be non-specific. Quantitative analyses showed that the total % of CADASIL microvessels stained by the complement factors in the WM (and grey matter) was 65-75% (Table 2). Specificity of the activation of the alternative pathway was demonstrated by the lack of complement factor C1q immunoreactivity and that C3d immunoreactivity was not apparent in PADMAL cases (Table 2; n = 3). We found that the same antibody labelled amyloid β deposits in AD cases (data not shown). To corroborate the *in-situ* findings, we demonstrated that C3d and Factor B were both immunolocalised in cultured VSMCs carrying the *NOTCH3 Arg133Cys* mutation from CADASIL subjects (Fig. 7). We calculated that the percentage of C3d + cells was 71.4, the percentage of Factor B + cells was 72.2 and the percentage of percentage of N3ECD + cells was 64.0 (n = 3 wells). Specificity was further shown by the localization of N3ECD immunoreactivity in CADASIL but not in control VSMCs. There was no apparent localisation of complement in the close proximity to N3ECD reactivity, which was at the outer cell membrane [57].

**Table 3:** Proteomic Studies in CADASIL

Study (Reference)	CADASIL (no); *NOTCH3* Mutation	Post-mortem Findings (no cases)	Sample type	Proteomic Methods	Changes in key identified proteins
Unlu et al., 2000 [59]	*Arg133Cys* (1); *Arg141Cys* (2);	SVD, WMD, lacunes (3)	CSF	2DGE, IEF, Mass Spectrometry	Complement Factor B, albumin (not consistently changed)
Ihalainen et al., 2007 [23]	*Arg133Cys* (3);	ND	Human VSMCs	2DGE, IEF, MALDI-TOF MS	RhoGDI, HSP27, profilin 1, CRP1, MnSOD, GST P1, GST omega
Arboleda-Velasquez et al., 2011 [2]	*Cys455Arg* (1); *Arg1031Cys (1)*	ND	Brain vessels isolated by LMD	LCM MS	Clusterin, endostatin
Monet-Lepretre et al., 2013 [33]	*Arg110Cys (1), Arg133Cys (1), Arg153Cys (1), Arg169Cys (1) Cys1261Arg (1)*	SVD, WMD, lacunes (5)	Isolated brain arteries (fractionation)	Trypsin digestion; nano LC-MS/ MS system	TIMP3, vitronectin
Nagatoshi et al., 2017† [35]	*Arg75Pro* (1); *Arg133Cys* (3); *Arg182Cys* (1)	SVD, WMD, lacunes (1)	Perivascular brain tissues by LMD	Trypsin digestion; Capillary reversed phase LC-MS/ MS system	Serum amyloid P component (SAP), annexin A2, periostin, Complement C3
Zellner et al., 2018 [66]	*Arg110Cys (1)* *D239-D253del (1)* *Cys144S (1)* *Arg153Cys (2)* *Cys1261Arg (1)*	SVD, WMD, lacunes (6)	Isolated CMV incl. capillaries	Trypsin digestion; LC-MS/ MS system	Mitochondrial proteins, HTRA1, COL6, COL8, COL12, SAP, TIMP3, vitronectin, clusterin, HTRA1, Complement C3
Present Study	*Arg133Cys* (2); *Arg169Cys* (2);	SVD, WMD, lacunes (4)	Isolated CMV incl. capillaries	Trypsin digestion of RIPA soluble fractions; nano LC-MS/ MS system	Several heat shock proteins (Fig. 3; Supplementary Table 1)

Table Notes: Matrix-assisted laser desorption ionization time of flight mass spectrometer (VOYAGER DE-STR). Mildly elevated protein levels found in CSF of CADASIL patients were ascribed to breakdown of the blood-CSF barrier [10]. Endothelial cells cultured from human brain microvessels are able to synthesise factor B locally in response to interferon gamma but not to serine proteases, plasmin, or miniplasmin [60]. †Serum SAP concentrations of CADASIL patients and healthy controls did not differ statistically

### Complement factors and the NOTCH3 signalling pathway

To investigate whether the increased complement in CADASIL was regulated by NOTCH3 signalling (supplementary results), we examined the regulation of these components by qRT-PCR in a NOTCH3 co-culture cellular system. qRT-PCR results showed no correlation between transcriptional activities of various components of the complement cascade pathway with the NOTCH3 signalling pathway. There was constitutive expression of complement factor B, C3, C5 and C9 in SH-SY5Y neuroblastoma cell lines stably expressing the NOTCH3 receptor. However, C1q was not expressed in the SH-SY5Y cell lines. SH-SY5Y cells, stably transfected with either wild type *NOTCH3* cDNA or p.*Arg133Cys* or p.*Cys185Arg* mutant *NOTCH3* cDNA showed no differences in expression level of complement factor B, C3, C5 and C9 (Supplementary Fig. 9). In addition, activation of the NOTCH3 receptor by a co-culturing system with ligand expressing HEK 293 cells, did alter the regulation of complement factors assayed suggesting their transcription activity was not affected by NOTCH3 signalling pathway activation (Supplementary Fig. 3). Increased immunoreactivity of complement factors observed within arterioles in CADASIL was not related to NOTCH3 signalling activation but suggested another mechanism. We tested whether VSMC degeneration could be due to apoptosis, caused by down regulation of c-FLIP and induction of Fas via an aberrant or mutant NOTCH3 signalling pathway. We utilised the same co-culture system to demonstrate that there was no effect on the regulation of NOTCH3 signalling on c-FLIP expression (Supplementary Fig. 9).

## Discussion

We have provided collective evidence to show that there is a robust inflammatory component in CADASIL, unlike that in sporadic cerebral SVD or in normally ageing subjects. Our findings suggest that the aggressive nature of the arteriopathy in CADASIL, characterised by profound loss of VSMCs, induces these responses which likely contribute to the progression of disease in many patients. The disruption of the ER by mutant NOTCH3 appears the main instigator of the inflammatory responses rather than necessarily the accumulation of GOM.

In this study, we specifically showed that (1) various cytoskeletal markers and chaperone proteins reveal profound abnormalities and subsequent loss of VSMCs within arterioles, (2) there is an ER stress response within microvessels as demonstrated by proteomic and immunolocalization analyses, (3) there is a specific perivascular inflammatory cell response characterised by the presence of greater densities of CD45^+^ and CD163^+^ cells, (4) VSMCs release several pro-inflammatory cytokines and integrins of IL-6 and ICAM-1, and (5) consistent with several previous studies indicating activation of the alternative complement pathway in 80–90% of chronic diseases [48], mediators of the alternative complement cascade were localised in VSMCs of CADASIL subjects.

Comparable with our observations, VSMCs within arterial walls of CADASIL patients become frail and assume even lower proliferation rates likely due to impaired mitotic activity [12, 46] even in isolated cells [23, 56]. Our pathological findings also showed that expression of various proteins associated with protein degradation, and antioxidant and heat shock responses are variably increased in CADASIL. Neves et al. [36] showed that ER stress target genes were amplified plus ER stress response, Rho kinase activity, superoxide production, and cytoskeleton-associated protein phosphorylation were increased in CADASIL. The responses involving upregulation of Nox5 were also recapitulated in TgNotch3 p.*Arg169Cys* mice. These changes are further compatible with spontaneous formation of oligomers and higher order multimers of N3ECD [40] that result in disruption of the ER and orderly presentation of NOTCH3 receptor at the cell surface.[56] In accord with this, our observations are in general compatible with previous findings from several proteomic studies (Table 3) which reported different proteins associated with the cytoskeletal and extracellular matrix structures, mitochondrial function [61] and immune responses (Table 3). However, we did find some dissimilarities in proteomic changes in isolated cerebral microvessels in our cases. We could not detect components of the complement or serum amyloid P in the proteomic analyses, yet these were readily and specifically detected by immunohistochemistry. We propose that differential progression in vascular pathogenesis and different isolation, extraction and detection methods depending on specific protein abundance in cellular compartments could account for some of the divergent observations. Nevertheless, several studies in other organ systems show that ER stress via NF-κB activation can either promote or impede disease progression via inflammatory pathways depending on cell type [18].

One of our key findings relates to the robust inflammatory response. We found increased perivascular inflammatory cells, particularly CD45 + microglia in CADASIL [45]. These cells did not appear to contain N3ECD immunoreactivity suggesting that they are specially activated because of degenerating vessel wall elements or other inflammatory activators associated with the vessels. The profound increased expression of ICAM-1 and IL-6 in vitro in VSMCs also supports the hypothesis that there is a unique inflammatory response in many arterioles in CADASIL. It is plausible that cytokines such as increase IL-6 function to recruit microglia/macrophages in degenerating vessels. The frailty of VSMCs and inflammation could promote fibrosis too, which is a characteristic for this disease much more in the WM than the grey matter [32].

Consistent with the first evidence we reported on the involvement of complement in CSF [59], we have now clearly shown that the alternative complement pathway is activated in CADASIL. To characterise the relation between activation of the complement system and the vasculopathology, we assessed key components of the complement pathway cascade in CADASIL. Our findings indicated specific immunoreactivity of complement factors C3d, C5-9 and B. The presence of C3 has been previously variably reported in CADASIL [6, 37, 47] and is apparent in proteomic studies (Table 3). However, this finding was not explored further. We also showed that such patterns of complement reactivity were not present in OC or SVD or PADMAL cases. The strongest staining intensity was unsurprisingly of complement factor C3d in 75% of the vessels whereas 65% and 70% of C5-9 and B, respectively. This suggests that the complement pathway is not activated in all vessels, or it could be that amounts present did not enable detection. Irrespective, we like others [6] could not detect complement C1q in CADASIL or YC although one earlier study had reported the presence of C1q in a single case [37].

We also showed that in VSMCs there was specific localisation of both Factor B and C3 but not in direct association with N3ECD immunoreactivity. Besides N3ECD deposits [25, 65], VSMCs have also been shown to produce complement Factor B and C3 in other vascular conditions [13, 29, 52, 53, 60] However, the distinctive immunostaining patterns observed in the CADASIL suggests that the complement system could play an important role in the vasculopathogenesis of the disorder. Perhaps more intriguing is the finding that only the alternative complement pathway is involved rather than the classical pathway. The presence of complement factors C3d and C5-9 also suggest that the complement pathway is fully activated with the formation of the membrane attack complex (C5-9), which indicates VSMCs may be vulnerable to further destruction via bystander lysis, inherent of inflammatory responses.

Although the activation of the complement pathway and inflammation could be the cause of degeneration of VSMCs, apoptosis is another possibility of such cellular death. Wang et al. [63] suggested that NOTCH3 signalling in VSMCs results in the induction of cellular c-FLIP expression via the ERK/MAPK activation. C-FLIP functions as an anti-apoptotic factor by inhibition of death receptor signals. We found that NOTCH3 activation did not result in any transcriptional regulation of c-FLIP. In addition, CADASIL causing mutations, p.*Arg133Cys* and p.*Cys185Arg*, did not disrupt or inhibit expression of c-FLIP.

To further investigate the possible involvement of NOTCH3 signalling with the activation of the complement pathway cascade, the transcription activity of various complement components was examined in an in vitro NOTCH3 cell culture system. We found no evidence to suggest that the transcriptional activity of complement factors was dependent or regulated by NOTCH3 signalling. The increased complement in arteries and arterioles of CADASIL patients is derived and upregulated independently of NOTCH3 signalling and largely explained by degeneration of VSMCs via another mechanism. It is conceivable that the increased complement immunoreactivity in CADASIL could result or is indirectly catalysed by aggregated N3ECD rather than GOM since VSMC degeneration appears to precede GOM deposition [49].

The main limitation of our study is perhaps the lack of ultrastructural localisation of complement in CADASIL brain tissues or the isolated VSMCs. Nevertheless, complement immunoreactivity was sufficiently strong in the media of many arteries and clear by double immunofluorescence in VSMCs from a patient with the p.*Arg133Cys* mutation but not in YC. Our proteomic analysis could have also been performed in a larger number of samples and using more sensitive detection methods, but we felt there are now several proteomic studies, which provide good evidence of the changed characteristics of the arteriopathy in CADASIL likely involving multiple factors (Table 3). Immunohistochemical experiments to demonstrate localisation of more proteins, will be explored in future studies.

## Conclusions

Our data support the role of inflammation and in particular the activation of the alternative complement pathway in CADASIL. Our observations suggest complement activation occurs to be independent of mutant NOTCH3 signalling. The complement activation and the inflammatory responses likely instigate further degeneration of VSMCs and promote the characteristic arteriopathy commonly observed in CADASIL. Given there are low-grade localised inflammatory responses in CADASIL, therapeutic measures that can reduce or abate these with e.g. non-steroidal anti-inflammatory agents could be beneficial for CADASIL and CADASIL-like patients. Other approaches including active immunisation may also be useful to explore to reduce the effects of N3ECD deposition and GOM in CADASIL [39]. This is important because there are limited strategies to improve the quality of life in these patients with the most common genetic form of SVD.

## Electronic supplementary material

Below is the link to the electronic supplementary material.

Supplementary Material 1

Supplementary Material 2

Supplementary Material 3

Supplementary Material 4

Supplementary Material 5

Supplementary Material 6

Supplementary Material 7
