# Supplementary material for: ER stress induced immunopathology involving complement in CADASIL: implications for therapeutics
Source: Acta Neuropathol Commun. 2023 May 8;11:76. doi: 10.1186/s40478-023-01558-1 (PMC10169505; doi:10.1186/s40478-023-01558-1)
Supplement: Supplementary file 1 — Supplementary Material 1 [file 40478_2023_1558_MOESM1_ESM.doc]

**Supplementary File 1: ER Stress Induced Immunopathology involving Complement in CADASIL: Implications for Therapeutics**

Mahmod Panahi^1*^, Yoshiki Hase,^2*^ Xavier Gallart-Palau,^3^ Sumonto Mitra,^1^ Atsushi Watanabe,^4^ Roger C Low,^2^ Yumi Yamamoto,^5^ Diego Sepulveda-Falla,^6^ Atticus H Hainsworth,^7^ Masafumi Ihara,^5^ Siu Kwan Sze,^8^ Matti Viitanen^1,9^, Homira Behbahani,^1^ Raj N Kalaria^2†+^

**Supplementary Figure 1**:

**Supplementary Figure 1:** **Bulk Isolation of Cerebral Microvessels for Proteomic Analysis.** Brain tissues from CADASIL cases and controls were homogenised in PBS containing protease inhibitors, and cerebral microvessels (CMV) were purified using glass bead columns [1]. To verify purity of CMV, fractions were smeared on glass slides, allowed to dry and stained with tinctorial stained such as toluidine blue or with antibodies to Von Willebrand Factor (VWF), the marker of vascular endothelial cells. CMV collected from glass bead columns were also immunostained with NOTCH3 antibodies N2 that recognized the extracellular domain (N3ECD). Compared CMV factions from CADASIL cases, controls revealed only background staining (N2 second row in panel). Moreover, the reactivities were not distinct with antibody C2 that recognized the intracellular domain of NOTCH3. For proteomic studies purified CMV were solubilized with RIPA buffer.

**Supplementary Figure 2**


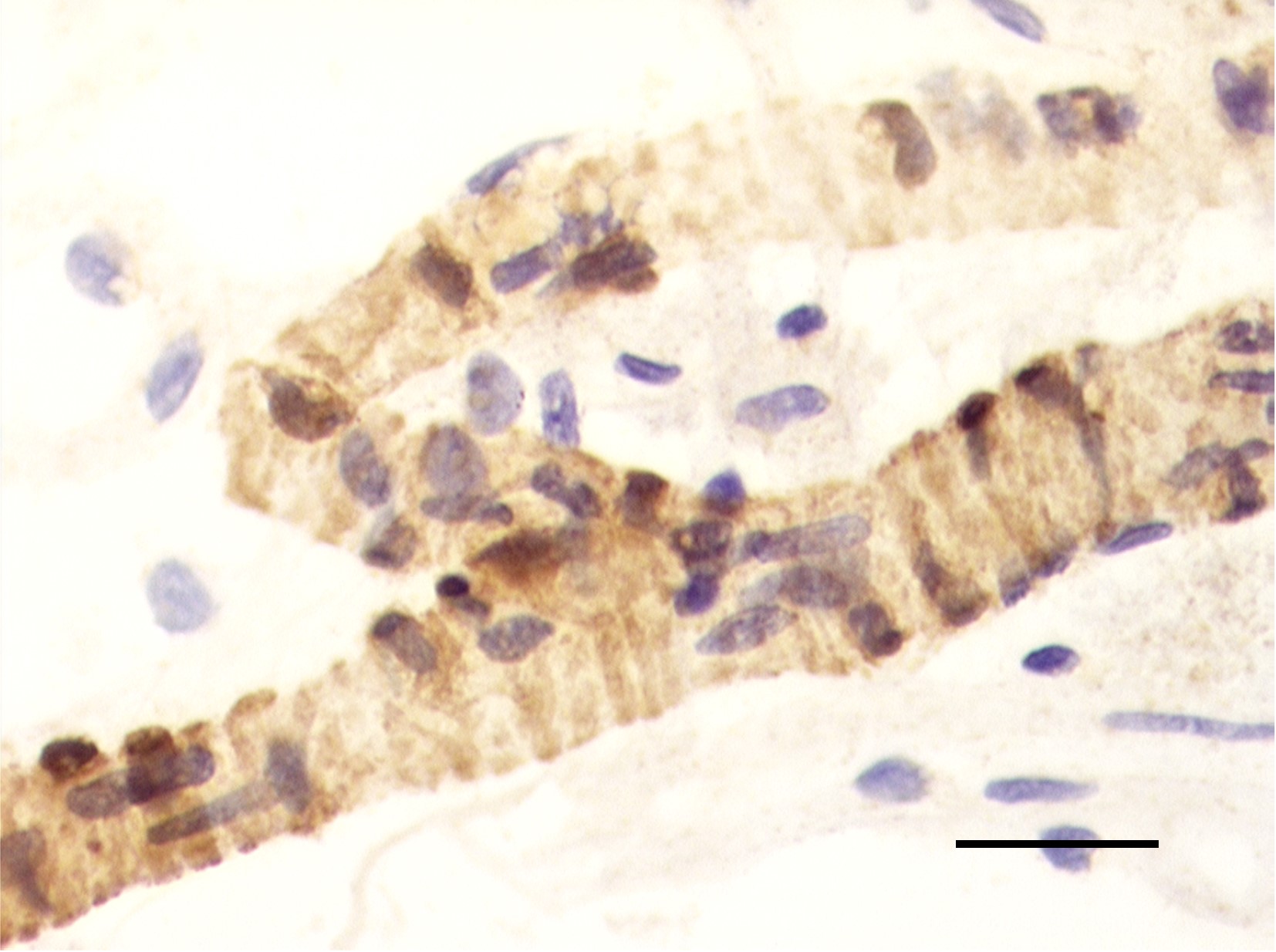


**Supplementary Figure 2:** Figure shows microvessel fragment derived from bulk isolation methods (Suppl Figure 1; cf. panel CADASIL, C2). Microvessels were also immunostained with α-SMA; concentric immunostained smooth muscle (SM) profiles were noted. Magnification bar: 50 μm

**Supplementary Figure 3**

**
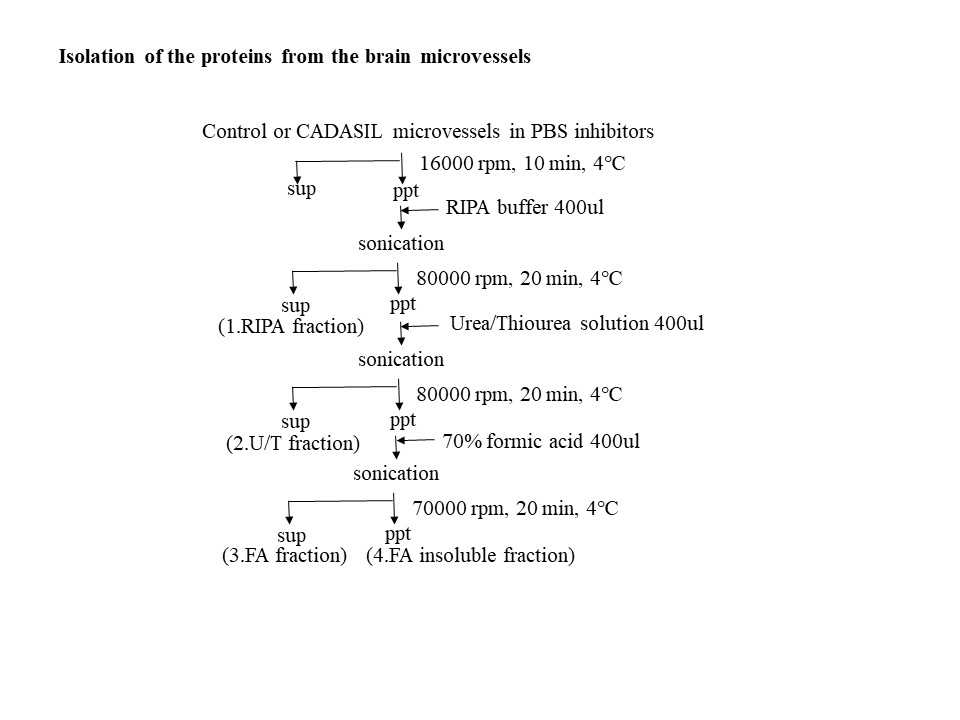
**

**Supplementary Figure 3: Protocol for isolation of proteins** Three different fractions were derived from original microvessels for analysis (See Methods).

**Supplementary Figure 4**


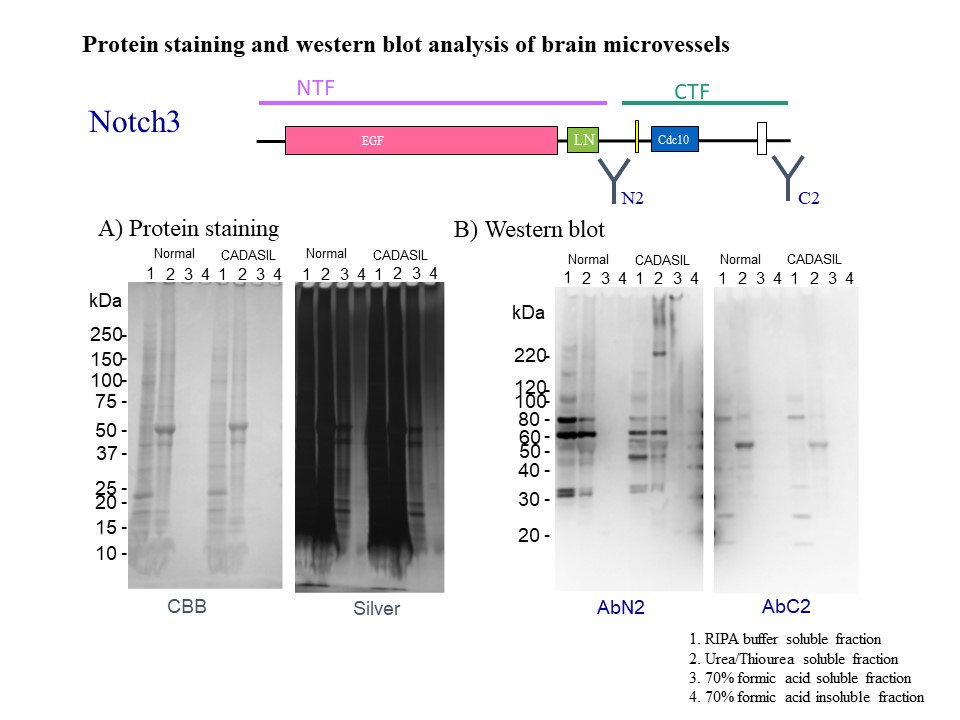


**Supplementary Figure 4: Protein and Immunoblotting gels shows specificity of the NOTCH3 antibodies.** Relevant immunoreactive bands show positivity to A1-1 (N3ECD) and N2 antibodies in different fractions of cerebral microvessel to localise NOTCH3 protein.

**Supplementary Figure 5:**


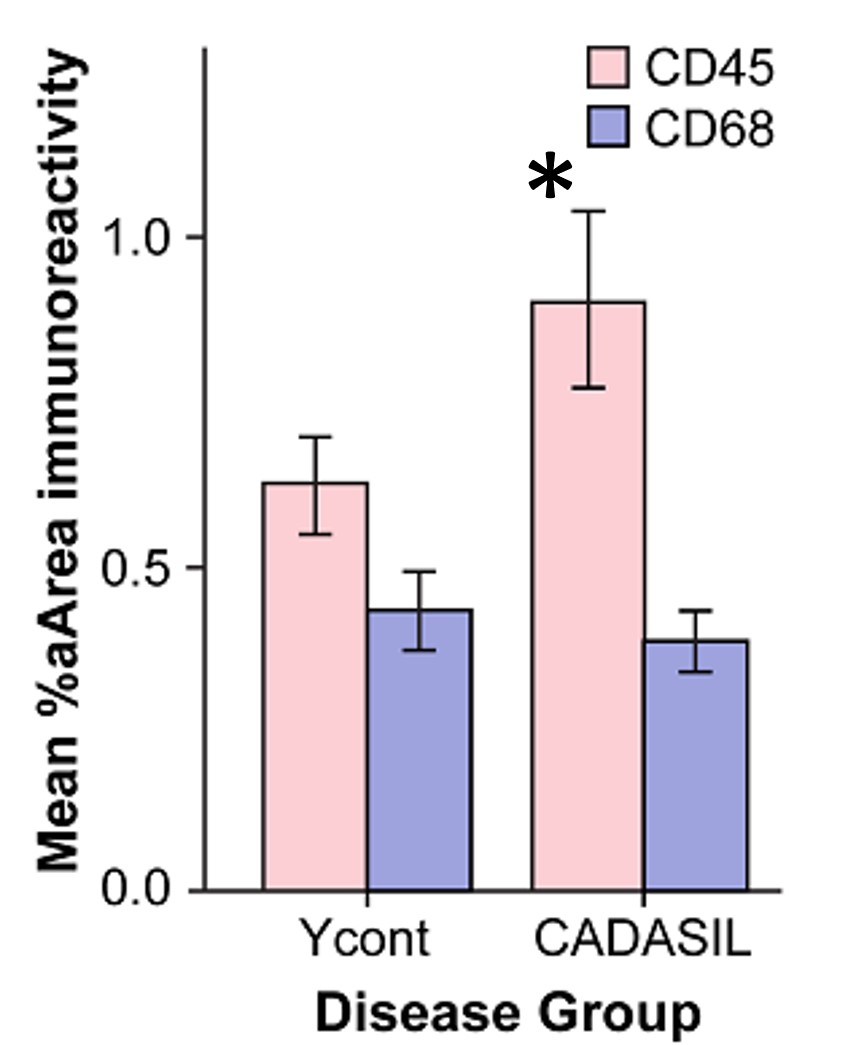


**Supplementary Figure 5: Quantification of Perivascular Inflammatory Cells.** Figure shows histograms with quantification of the % specific area of CD marker cells in CADASIL and similar age controls (Ycont). Mean +SEM from n=9 cases each. * There were greater number of rounded (active) CD45+ve cells compared to CD68+ve cells (P<0.05).

**Supplementary Figure 6:**


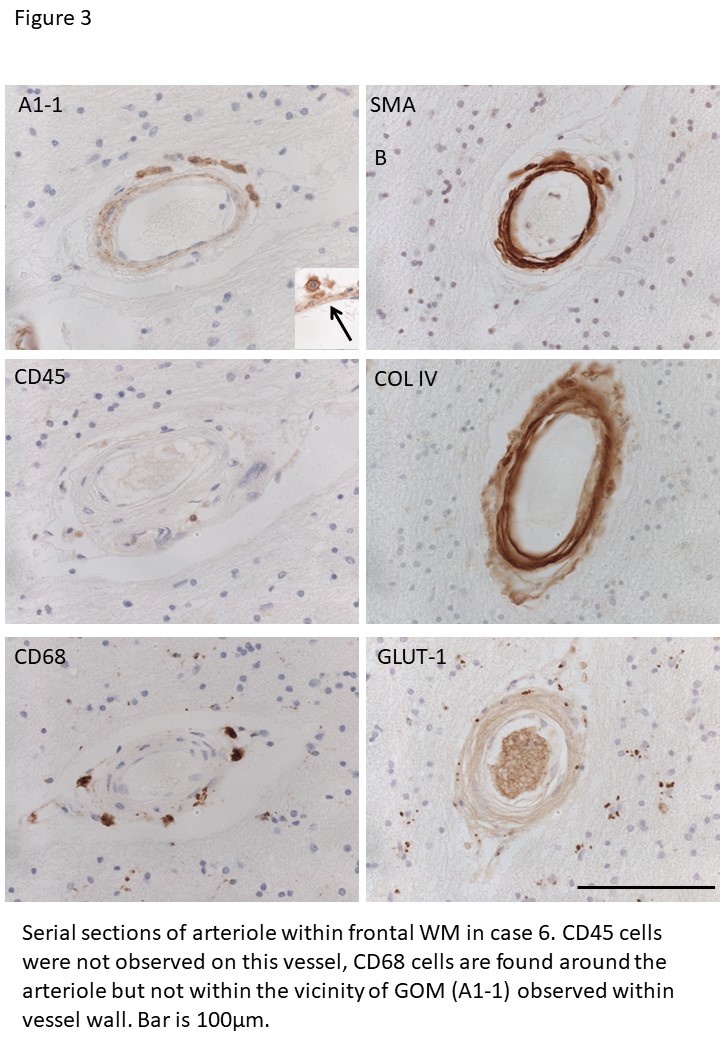


**Supplementary Figure 6:**  **Differential localisation of N3ECD containing cells, CD45 and CD 68 cells.** Figure shows serial sections of an arteriole within frontal WM in a CADASIL case (CAD6, Table 1). CD45^+^ cells were not observed on this vessel, some CD68 cells are found around the arteriole but not within the vicinity of N3ECD (A1-1) observed within vessel wall. If N3ECD was accumulated by CD45 or CD68 cells they would be localised perivascularly. Inset in A1-1 (N3ECD), shows a cell immunostained for N3ECD. Magnification bar represents 100µm.

**Supplementary Figure 7:**


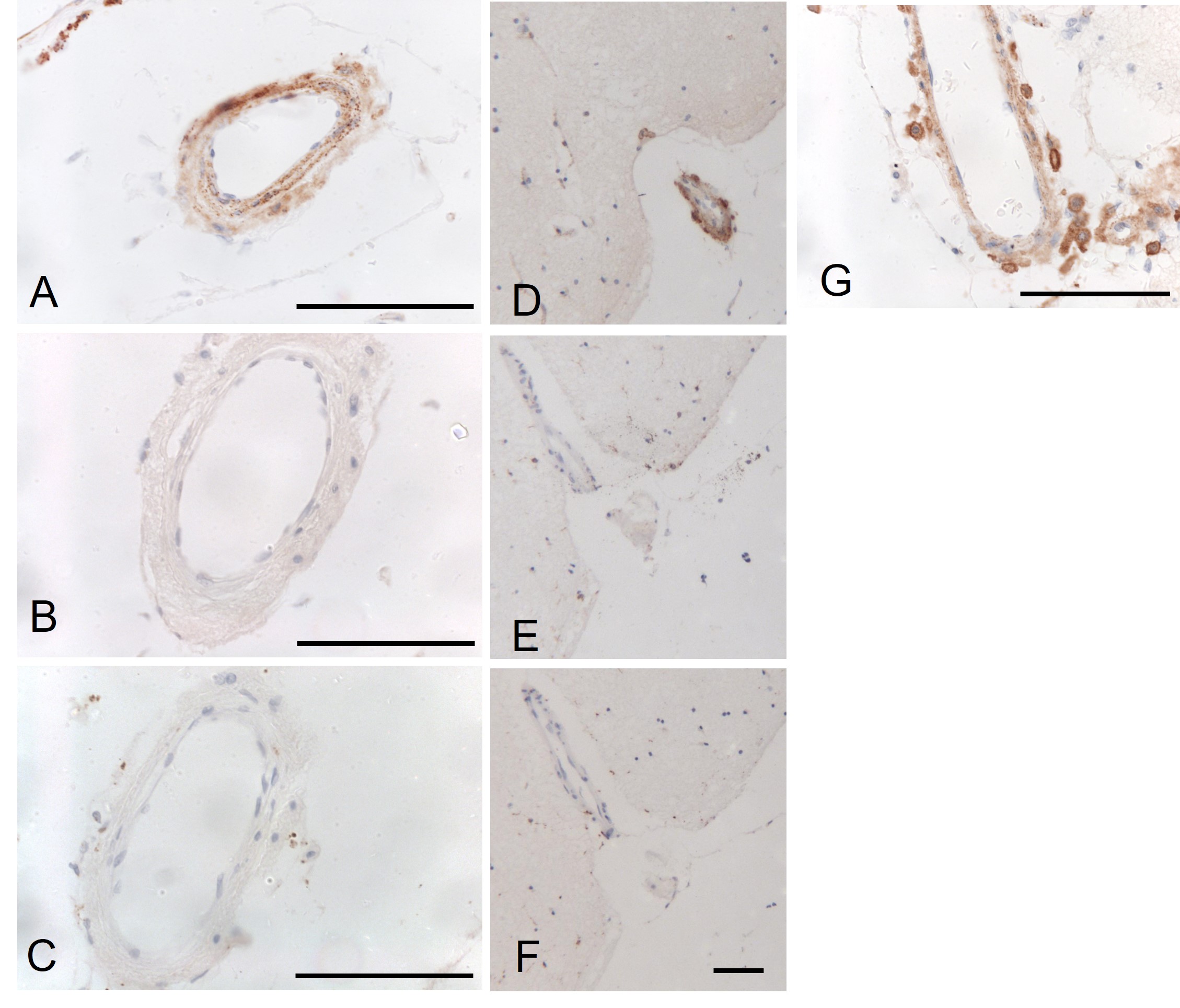


**Supplementary Figure 7: Differential localisation of N3ECD containing cells, CD45 and CD 68 cells.** Figure shows serial sections of subarachnoid arterioles within the frontal lobe in two CADASIL cases (A-C and D-G) (Table 1). CADASIL Case 6 (A-C): Meningeal arteriole above frontal cortex has N3ECD +ve deposits but same vessel did not have CD45 +ve or CD68 +ve cells or any similarly distributed as N3ECD (A). CADASIL case 11 (D-G) Meninges above frontal cortex stained for N3ECD in meninges, in macrophage-like or clasmatodendritic cells [4] can be seen around blood vessel and stain positively for N3ECD (D, G) Staining for CD45 shows these cells are not present when CD45 (E) or CD68 (F) is used as a marker. Magnification bars represent 100µm (A-C, D-F, G) .

**Supplementary Figure 8:**


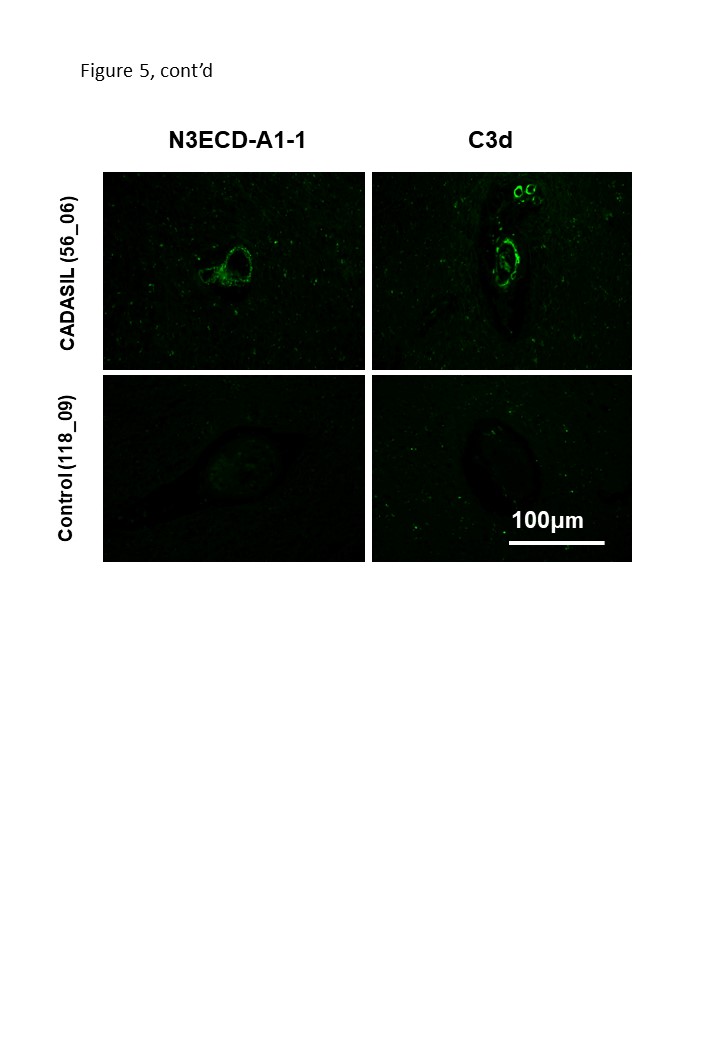


**Supplementary Figure 8: Localisation of C3d by immunofluorescence (IF) in CADASIL.** Figure shows in situ localization of C3d immunoreactivity by IF in CADASIL but not control subject.

**Quantification of complement factor B, C3, C9 and anti-apoptotic marker c-FLIP in a co-culture system by quantitative reverse transcription polymerase chain reaction (Q-RT-PCR)**

**Methods**

*Generation of Stable SH-SY5Y Neuroblastoma Cell Line Expressing Notch3 Receptor.*

*NOTCH3* cDNA of both wild type and gain of cysteine mutant, R133C or loss of cysteine mutant, C185R [2, 3], were transfected into SH-SY5Y with Effectene according to manufacturer instructions (Qiagen). Briefly, DMEM/F12 (Gibco) supplemented with 10% fetal calf serum (Gibco) and 1% penicillin/ streptomycin antibiotics (Gibco) non-selection medium was used for maintenance of SH-SY5Y cultures. Cells were seeded at 5x10^5^ cells the day period to transfection. 0.4μg of *NOTCH3* cDNA pCEP4 clone were used per transfection as according to manufacturer instructions. Fresh medium was added 24 hours after post-transfection and cells were incubated further for 24 hours before changing to selection medium of non-selection medium added with 400μg/ml hygromycin (Wako). Cells were maintained in selection medium where fresh medium was changed every other day initially for 1 week before reducing the frequency to once every 4 to 5 days.

Cells were further maintained in such manner until single colonies of resistance cells are formed. Single colony were isolated and allowed for further growth until 100% confluent in a 60mm culture plate. Cells were trypsinised with 1X Trypsin-EDTA solution (Gibco) and harvested. Harvested cells were split equally into 2 portions with 1 portion re-seeded in selection medium for further growth and the other half were used for Western Blot for detection of the expression level of transfected clone. Several clones of each *NOTCH3* type were assayed with either N1, N2 or C2 antibodies [2]. Colonies with high expression of the NOTCH3 receptor for both wild type and mutants were selected and maintained further in selection medium. Non-expressing colonies were discarded.

*NOTCH3 Receptor and Ligand Co-culture System*

Stable HEK 293T cell lines expressing either Jagged1 ligand or Delta1 ligand was kind gift of Drs Y. Santa and K. Takahashi (National Institute for Longevity Science, Obu, Japan). These ligands expressing stable HEK 293T cell lines were maintained in DMEM (Gibco) supplemented with 10% fetal calf serum, 1% penicillin/streptomycin and 100μg zeocin (Invitrogen). During co-culture, all medium used were devoid of any antibiotics except the routinely added 1% penicillin/streptomycin. Control cell lines of HEK 293T stably transfected with empty pTRACER-CMV2 expression vector (Invitrogen) were maintained in the same conditions and used for all control experiments.

Co-culture system was performed with firstly plating the stable SH-SY5Y cell lines expressing either wild type or mutant *Notch3* such that they will reach about 50-60% confluent the next day. Control cells of SH-SY5Y cell lines stably transfected with the pCEP4 empty control vector (Invitrogen) was similarly plated. Stable HEK 293T cell line over expressing the NOCTH3 ligand of either Delta1 or Jagged1 were added in equal number as the plated *NOTCH3* stable SH-SY5Y cell lines and co-culture in DMEM/F12 medium without any selection antibiotics. *NOTCH3* cell lines were similarly co-cultured with control HEK 293T cell lines as control experiments.

*RNA Extraction and cDNA Synthesis*

Stable SH-SY5Y NOTCH3 (wild type and mutants) cell lines (see chapter 5 for details of cell line generation) were plated to about 60-70% confluence. Stable HEK293 ligand (either Jagged1 or Delta1 cloned into pTracerCMV, Clontech) cell lines or control HEK293 stably transfected with pTracerCMV (Clontech) empty vector (all HEK293 stable cell lines were kind gifts of Drs Y. Santa and K. Takahashi, National Institute for Longevity Science, Obu, Japan) were added at half the original number of cells present to reduce the effect of transcriptional activities from the stable HEK293 ligand cell lines. Cells were co-cultured for three, six, nine or twenty-four hours before total RNA extraction.

All RNA extraction was performed using RNeasy Mini Kit, RNA extraction kit (QIAGEN) and [QIAshredder homogenizers](http://www1.qiagen.com/catalog/chapter_11/chap11f.asp) (QIAGEN) as according to manufacturer’s instructions. cDNA was synthesized from the extracted RNA using random hexamer primers provided in the Advantage RT-PCR kit (Clontech). All reverse transcription polymerase chain reactions conditions used were as according to manufacturer instructions (Clontech).

*Real Time Quantitative Reverse Transcription Polymerase Chain Reaction (Q-RT-PCR)*

Analysis of transcription activation upon Notch3 signalling activation with co-culture systems was performed with Quantitative RT-PCR (Q-RT-PCR) on LightCycler (Roche). Genes investigated are, complement factor B, complement factor C1qB, complement factor C3, complement factor C5, complement factor C9, c-FLIP and β-actin as housekeeping control gene. Oligonucleotide primers for all genes investigated were designed to be intron-spanning to differentiate between genomic DNA and cDNA amplification, whereby only products from cDNA amplification will be small enough for quantification on the LightCycler than those from genomic DNA amplification.

Primers for complement factor B were, 5’ AAG GTC AGC GTA GGA GGG GAG AAG 3’ and 5’ CAG CTG CCT TTC TTA TCC CCA TTC 3’; complement factor C1qB were, 5’ CCC AGG GAT AAA AGG AGA GAA AGG 3 and 5’ GGC GTG GTA GGT GAA GTA GTA GAG 3’; complement factor C3 were, 5’ TCA CCG TCA ACC ACA AGC TGC TAC C 3’ and 5’ TTT CAT AGT AGG CTC GGA TCT TCC A 3’; complement factor C5 were, 5’ GTG GCA TTA GCA GCA GTG GAC AGT G 3’ and 5’ GCA GGC TCC ATC GTA ACA ACA TTT C 3’; complement factor C9 were, 5’ GAA TGA GCC CCT GGA GTG AAT GGT C 3’ and 5’ CAT TTC CGC AGT CAT CCT CAG CAT C 3’; c-FLIP were 5’ ACA GTT CAC CGA GAA GCT GAC T 3’ and 5’ TCC TTG GCA GAA ACT CTG CTG T 3’; and β-actin were, 5’ CCA GGT CAT CAC CAT TGG CAA 3’ and 5’ ACA GGT CTT TGC GGA TGT CCA 3’.

All Q-RT-PCR were performed with cDNA synthesized (see section 4.2.8) on the LightCycler (Roche) with DNA master SYBR green I kit (Roche) according to manufacturer instructions. Annealing temperature used were 59^o^C for complement factor B, 55^o^C for complement factor C1qB, complement factor C3, complement factor C5 and complement factor C9, 61^o^C for c-FLIP and β-actin. Expected amplicons size were 295bp, 358bp, 186bp, 315bp, 180bp, 192bp and 144bp for complement factor B, C1qB, C3, C5, C9, c-FLIP and β-actin respectively.

Q-RT-PCR for all genes analysed were done in the recommended 40 cycles. Triplicate assay were performed for all the genes investigated and the mean value were calculated and normalised against those calculated from β-actin as control for concentration of cDNA loaded before further tabulated against the transcription activity of SH-SY5Y control cells stably transfected with the empty pCEP4 vector and co-cultured to the respectively 293 ligand stable cell lines (or controls) accordingly to determine the regulatory effects on these gene transcription activity upon wild type or mutant Notch3 signalling activation. Amplicons from Q-RT-PCR were extracted and ran on 2% agarose gel to determine the specificity of the amplification by the amplicon size.

**Supplementary Figure 9:**

**A**

**B**

**C**

**D**

**E**

**Supplementary Figure 9:** Quantification of complement factor B, C3, C9 and anti-apoptotic marker c-FLIP in SH-SY5Y neuroblastoma cell line stably transfected with either Notch3 wild type or mutant cDNA or empty pCEP4 control vector. There are no significant changes in the expression level of all the 4 genes investigated here between the wild type Notch3 cell line and mutant cell lines. In addition, transfection of the SH-SY5Y cell line with the Notch3 cDNA does not result in any changes in the expression level of all these genes. Data were firstly normalised with those obtained for house-keeping gene, α-actin and further normalised with those obtained from SH-SY5Y cells stably transfected with the empty control pCEP4 vector for fold increment of the respective genes assayed.

B-D. Quantification of complement factor B (B), factor C3 (C) and factor C9 (D) by Q-RT-PCR after 24 hours co-culture for ligand mediated Notch3 activation of both wild type and mutants Notch3 clones. Notch3 activation by either Jagged1 or Delta1 expressing cells appear not to affect the transcription regulation of all the 3 complement factors examined here in both Notch3 wild type and mutants cell line. In addition, transcription level of these complement factors is not significantly different from the expression level in control SH-SY5Y cell line stably transfected with empty pCEP4 vector.

E. Quantification of anti-apoptotic marker, c-FLIP by Q-RT-PCR after 24 hours co-culture for ligand mediated Notch3 activation of both wild type and mutants Notch3 clones. Expression level of c-FLIP is not affected by Notch3 activation by either Jagged1 or Delta1 expressing cells suggesting that Notch3 signalling pathway activation has no transcriptional regulation of the gene above the basal level normally observed in the SH-SY5Y neuroblastoma cell lines.

**Supplementary Table 1:** Untargeted label-free quantification of proteome abundance in CADASIL and Control samples. Proteins with spectral count >20 were included in the list. In CADASIL, several heat shock proteins were consistently increased compared to controls.

**References:**

1 Kalaria RN, Harik SI (1988) Adenosine Receptors and the Nucleoside Transporter in Human-Brain Vasculature. J Cerebr Blood F Met 8: 32-39 Doi Doi 10.1038/Jcbfm.1988.5

2 Low WC, Santa Y, Takahashi K, Tabira T, Kalaria RN (2006) CADASIL-causing mutations do not alter Notch3 receptor processing and activation. Neuroreport 17: 945-949 Doi 10.1097/01.wnr.0000223394.66951.48

3 Takahashi K, Adachi K, Yoshizaki K, Kunimoto S, Kalaria RN, Watanabe A (2010) Mutations in NOTCH3 cause the formation and retention of aggregates in the endoplasmic reticulum, leading to impaired cell proliferation. Hum Mol Genet 19: 79-89

4 Yamamoto Y, Craggs LJ, Watanabe A, Booth T, Attems J, Low RW, Oakley AE, Kalaria RN (2013) Brain microvascular accumulation and distribution of the NOTCH3 ectodomain and granular osmiophilic material in CADASIL. J Neuropathol Exp Neurol 72: 416-431 Doi 10.1097/NEN.0b013e31829020b5
